# Horizontal gene transfer in *Histophilus somni *and its role in the evolution of pathogenic strain 2336, as determined by comparative genomic analyses

**DOI:** 10.1186/1471-2164-12-570

**Published:** 2011-11-23

**Authors:** Shivakumara Siddaramappa, Jean F Challacombe, Alison J Duncan, Allison F Gillaspy, Matthew Carson, Jenny Gipson, Joshua Orvis, Jeremy Zaitshik, Gentry Barnes, David Bruce, Olga Chertkov, J Chris Detter, Cliff S Han, Roxanne Tapia, Linda S Thompson, David W Dyer, Thomas J Inzana

**Affiliations:** 1 Center for Molecular Medicine and Infectious Diseases, Virginia-Maryland Regional College of Veterinary Medicine, Virginia Polytechnic Institute and State University, Blacksburg, Virginia 24061, USA; 2 DOE Joint Genome Institute, Los Alamos National Laboratory, Los Alamos, New Mexico 87545, USA; 3 Laboratory for Genomics and Bioinformatics, and Department of Microbiology and Immunology, University of Oklahoma Health Sciences Center, Oklahoma City, Oklahoma 731042, USA

## Abstract

**Background:**

Pneumonia and myocarditis are the most commonly reported diseases due to *Histophilus somni*, an opportunistic pathogen of the reproductive and respiratory tracts of cattle. Thus far only a few genes involved in metabolic and virulence functions have been identified and characterized in *H. somni *using traditional methods. Analyses of the genome sequences of several *Pasteurellaceae *species have provided insights into their biology and evolution. In view of the economic and ecological importance of *H. somni*, the genome sequence of pneumonia strain 2336 has been determined and compared to that of commensal strain 129Pt and other members of the *Pasteurellaceae*.

**Results:**

The chromosome of strain 2336 (2,263,857 bp) contained 1,980 protein coding genes, whereas the chromosome of strain 129Pt (2,007,700 bp) contained only 1,792 protein coding genes. Although the chromosomes of the two strains differ in size, their average GC content, gene density (total number of genes predicted on the chromosome), and percentage of sequence (number of genes) that encodes proteins were similar. The chromosomes of these strains also contained a number of discrete prophage regions and genomic islands. One of the genomic islands in strain 2336 contained genes putatively involved in copper, zinc, and tetracycline resistance. Using the genome sequence data and comparative analyses with other members of the *Pasteurellaceae*, several *H. somni *genes that may encode proteins involved in virulence (*e.g*., filamentous haemaggutinins, adhesins, and polysaccharide biosynthesis/modification enzymes) were identified. The two strains contained a total of 17 ORFs that encode putative glycosyltransferases and some of these ORFs had characteristic simple sequence repeats within them. Most of the genes/loci common to both the strains were located in different regions of the two chromosomes and occurred in opposite orientations, indicating genome rearrangement since their divergence from a common ancestor.

**Conclusions:**

Since the genome of strain 129Pt was ~256,000 bp smaller than that of strain 2336, these genomes provide yet another paradigm for studying evolutionary gene loss and/or gain in regard to virulence repertoire and pathogenic ability. Analyses of the complete genome sequences revealed that bacteriophage- and transposon-mediated horizontal gene transfer had occurred at several loci in the chromosomes of strains 2336 and 129Pt. It appears that these mobile genetic elements have played a major role in creating genomic diversity and phenotypic variability among the two *H. somni *strains.

## Background

*Histophilus somni *is a commensal or opportunistic pathogen of the reproductive and respiratory tracts of cattle. *H. somni *was initially identified as the etiologic agent of bovine thrombotic meningoencephalitis (TME), but also causes bovine shipping fever pneumonia, either independently or in association with *Mannheimia haemolytica *and *Pasteurella multocida*. Pneumonia and myocarditis are currently the most commonly reported diseases due to *H. somni *[1]. Infections resulting in abortion, infertility, arthritis, septicemia, and mastitis can also be caused by *H. somni *with varying degrees of frequency and severity in cattle [2]. Similar disease conditions associated with strains of *H. somni *have been described in sheep [2]. Relatively less pathogenic and/or avirulent variants of *H. somni *have also been isolated from cattle, most frequently from the mucosal surfaces of the genital tract [3].

Numerous *in vitro *and *in vivo *studies during the pre-genomic era have shed light on the differences in virulence properties between *H. somni *pathogenic isolates from sick animals and serum-sensitive commensal isolates from the genital tract [4]. However, thus far only a few genes involved in lipooligosaccharide (LOS) biosynthesis and serum-resistance have been identified in *H. somni *using DNA/DNA and DNA/protein comparisons [5-7]. *H. somni *pneumonia strain 2336 and preputial strain 129Pt have been comprehensively characterized phenotypically and have been analyzed in several comparative studies [8-10]. However, a comprehensive understanding of the genetic basis that determines the phenotypic variability among *H. somni *stains is necessary to gain further insights into their pathogenicity.

Comparative (*in silico*) analysis of bacterial genomes is a powerful tool for the prediction and/or identification of biochemical differences, virulence attributes, pathogenic ability, and adaptive evolution among related species/strains [11]. Among the *Pasteurellaceae*, the genomes of one or more species pathogenic to humans or animals from the genera *Actinobacillus, Haemophilus, Mannheimia, Pasteurella*, and others have been sequenced. The availability of these genome sequences has facilitated whole genome comparisons that have provided insights into the physiology and pathogenic evolution of the corresponding bacteria [12,13].

Horizontal gene transfer (HGT: defined as the "acquisition of new genes either directly by transformation with naked DNA, transduction with phages, or the uptake of plasmids or chromosomal fragments by conjugation") plays a critical role in driving the evolution of pathogenic bacteria [14]. Reduction in genome size (referred to as reductive evolution) can occur as a result of continuous loss of genetic material due to gene deletion and/or mutation followed by DNA erosion [15]. Previous analyses by biochemical and pulsed field gel electrophoresis indicated that *H. somni *strains 2336 and 129Pt have common ancestry, but are non-clonal [16,17]. The following mechanisms may have engendered the genetic differences between these strains: (i) only one strain acquired genes by HGT while the other one did not; (ii) only one strain lost genes by deletion/mutation and underwent '*reductive evolution*'; (iii) both strains independently and continuously acquired and lost genes, and the net loss or gain of genes is a determinant of their divergent evolution; (iv) gene convergence and the accumulation of synonymous and/or nonsynonymous nucleotide substitutions occurred across the genomes of the two strains.

The rationale for the present study was to determine, using whole genome sequencing and comparative genomics, the mechanisms responsible for genetic variability between the two strains. It was also envisaged that a comparative genomics and bioinformatics approach would facilitate identification of *H. somni *genes putatively involved in virulence and pathogenesis.

## Methods

Genomic DNA (2 mg) from *H. somni *strain 2336 was purified using the Puregene protocol (Gentra Systems, Minneapolis, MN). The shotgun sequencing phase for this genome required ~35,200 sequence reads to reach 8-fold coverage [18]. Library construction, template preparation, sequencing, assembly, and data analyses were performed as described previously [19,20]. The sequence data assembled with Phred-Phrap were viewed using Consed to assess data quality and design closure experiments. Consed was also used to identify putative repeat regions so that the problems associated with assembling these regions could be resolved by way of combinatorial PCR experiments to isolate the repeat sequences on PCR amplicons. The location and exact sequence of each repeat was confirmed by isolating PCR fragments that contained each repeat in its entirety, followed by primer walking across the PCR product.

For initial gap closure, Single Primer Amplification of Contig Ends (SPACE), which is similar to the single-primer PCR procedure for rapid identification of transposon insertion sites, was used [21]. Additional primers were designed, as necessary, to verify the correct assembly of contigs by confirmatory PCR. Simultaneously, a fosmid library was constructed for scaffolding purposes using the vector pCC1fos (Epicentre Biotechnologies, Madison, WI) with 40 kb inserts. Sequencing of the fosmids was necessary to close gaps across sequences that occur more than once in the genome, such as those of insertion sequences and ribosomal genes. Gaps that were not closed by SPACE-walking were closed using the sequence of *H. somni *strain 129Pt as a scaffold and the reads were assembled with parallel phrap (High Performance Software, LLC). Gap closure at this stage was also facilitated by AUTOFINISH [22]. Possible mis-assemblies were corrected with Dupfinisher [23] or transposon bombing of bridging clones [24] using an EZ::TN™ kit (Epicentre). The National Human Genome Research Institute standards for the Human Genome Project (1 error per 10, 000 assembled bases) were followed for *H. somni *to obtain sufficient quality genomic data.

Final automated annotation of the genome of strain 2336 was performed at the Oak Ridge National Laboratory using methods similar to those used to annotate the strain 129Pt genome [13]. Briefly, protein domains were identified by comparing each predicted protein against a Hidden Markov Model protein family database [25]. To estimate the number of proteins specific to each strain, the Smith-Waterman algorithm [26] was used to compare all predicted proteins from strain 129Pt against those from strain 2336 and vice-versa. Proteins deemed to be specific to each strain were compared against the NCBI non-redundant protein database to determine whether they were hypothetical or conserved hypothetical. The translated ORF was named a hypothetical protein if there was less than 25% identity or an aligned region was less than 25% of the predicted protein length. Prediction of the number of subsystems and pairwise BLAST comparisons of protein sets within strains 2336 and 129Pt were carried out with the Rapid Annotation using Subsystems Technology (RAST), which is a fully automated, prokaryotic genome annotation service [27]. This platform identifies tRNA and rRNA genes using the tools tRNAscan-SE and "search_for_rnas", respectively [27].

Multiple genome comparisons were performed using the 'progressive alignment' option available in the program MAUVE version 2.3.0 [28,29]. Default scoring and parameters were used for generating the alignment. A synteny plot was generated using the program NUCmer, which creates a dot plot based on the number of identical alignments between two genomes [30]. Prophage regions (PRs) were identified using Prophinder http://aclame.ulb.ac.be/Tools/Prophinder/, an algorithm that combines similarity searches, statistical detection of phage-gene enriched regions, and genomic context for prophage prediction [31]. Identification and annotation of genomic islets/islands (GIs) other than prophages were performed based on sequence composition bias and comparative genomic analysis [32]. Briefly, differences in the GC content, the occurrence of 'cornerstone genes' (*e.g*., transposases), and/or a continuous stretch of genes encoding hypothetical proteins were used as reference points for detection of GIs. Insertion sequences (ISs) were identified by whole genome BLASTX analysis of strains 2336 and 129Pt using the IS finder http://www-is.biotoul.fr/. Gene acquisition and loss among the two strains was determined by comparing gene order, orientation of genes (forward/reverse), GC content of genes (percent above or below whole genome average), features of intergenic regions (*e.g*., remnants of IS elements, integration sites), and the similarity of proteins encoded by genes at a locus of interest (> 90% identity at the predicted protein level). Putative horizontally transferred genes (HTGs; defined as genes whose greatest homology, based on BLASTP scores, is to genes from a more distant phylogenetic group than to genes from the same or a close phylogenetic group as the query genome) were compiled using the integrated microbial genomes (IMG) system http://img.jgi.doe.gov. This system uses not only the best hit (*i.e*., the homolog with the highest bitscore), but also all the matches that have bitscores equal to or greater than that of the best hit to identify putative HTGs [33]. DNA and protein sequences were aligned using the ClustalW http://www.ebi.ac.uk/Tools/clustalw2/index.html and BOXSHADE http://www.ch.embnet.org/software/BOX_form.html programs as described previously [34].

## Results

### Properties of the chromosomes

The size of the *H. somni *strain 2336 chromosome was 2,263,857 bp, and was larger than the chromosomes of the related bacteria *Haemophilus ducreyi *strain 35000HP (1,698,955 bp), *Haemophilus influenzae *strain Rd KW20 (1,830,138 bp), *H*. *influenzae *strain 86-028NP (1,913,428 bp), *H. somni *strain 129Pt (2,007,700 bp), *Neisseria gonorrhoeae *strain FA 1090 (2,153,922 bp), *Neisseria meningitidis *serogroup A strain Z2491 (2,184,406 bp), and *P. multocida *strain Pm70 (2,257,487 bp). However, the chromosome of *H. somni *strain 2336 was smaller than that of *Haemophilus parasuis *SH0165 (2,269,156 bp), *N*. *meningitidis *serogroup B strain MC58 (2,272,360 bp), *Actinobacillus pleuropneumoniae *L20 (2,274,482 bp), *Aggregatibacter aphrophilus *NJ8700 (2,313,035 bp), *Mannheimia succiniciproducens *strain MBEL55E (2,314,078 bp), and *M. haemolytica *strain BAA-410 (~2,569,125 bp, draft sequence). Although the chromosomes of the two *H. somni *strains differ in size by ~256,000 bp, their average GC content, gene density (total number of genes predicted on the chromosome), and percentage of the sequence (number of genes) that encodes proteins were similar (Table 1). *H. somni *strain 2336 did not contain plasmids, but *H. somni *strain 129Pt contained a single plasmid [34]. Some of the other relevant features of the chromosomes of *H. somni *strains 2336 and 129Pt are shown in Table 1.

**Table 1:** Characteristics of the genomes of *H.somni *strains

Genome	Strain 2336	Strain 129Pt
Chromosome size	2263857 bp	2007700 bp
Number of protein coding genes	1980	1792
Overall coding density	88.56%	89.6%
*Number of subsystems	247	240
G+C content	37.38%	37.19%
5S ribosomal RNA genes	6	6
16S ribosomal RNA genes	5	5
23S ribosomal RNA genes	5	5
Number of tRNA genes	49	49
Plasmids	None	pHS129 (5178 bp) [34]
Prophage regions	4	1
Genomic islands	3	6
GenBank accession number	[GenBank:CP000947]	[GenBank:CP000436]

*Subsystems predicted by RAST server [27].

**Figure 1:**
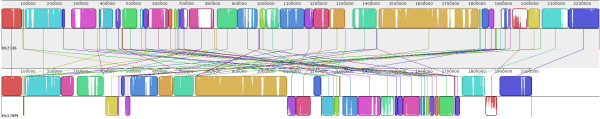
**Alignment of the chromosomes of strains 2336 (top) and 129Pt (bottom) using MAUVE 2**. Identically colored boxes, known as locally collinear blocks (LCBs), depict homologous regions in the two chromosomes. The edges of LCBs indicate chromosome rearrangements due to recombination, insertions, and/or inversions. Sequences of strain 129Pt inverted in relation to those of strain 2336 are shown as blocks below the horizontal line. The vertical lines connect regions of homology among the two chromosomes. Numbers above the maps indicate nucleotide positions within the respective chromosomes.

**Figure 2:**
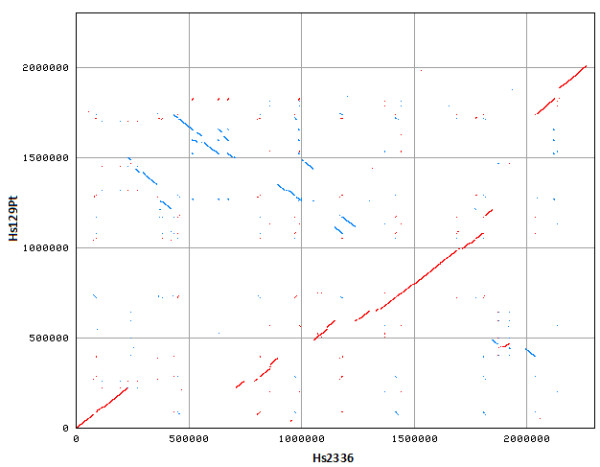
**Synteny plot of the chromosomes of strains 2336 and 129Pt generated by NUCmer**. Regions of identity between the two chromosomes are shown based on pair-wise alignments. The strain 2336 sequence is represented on the X-axis, and the strain 129Pt sequence is represented on the Y-axis. Numbers indicate nucleotide positions within the respective chromosomes. Plus strand matches are slanted from the bottom left to the upper right corner and are shown in red. Minus strand matches are slanted from the upper left to the lower right and are shown in blue. The number of dots/lines shown in the plot is the same as the number of exact matches found by NUCmer.

Whole genome alignment using MAUVE showed the presence of extensive blocks of homologous regions, which is typical of closely related genomes (Figure 1). To further dissect their co-linearity, a BLASTN comparison of the two genomes was performed at the Joint Genome Institute web site. This analysis indicated that there were 400 homologous regions (219 plus/plus and 181 plus/minus, sequence range > 1,000 bp, but < 30,000 bp) and the average nucleic acid identity among these homologous regions was 98.5% (the identity range was 94%-99%, E-value = 0). The plus/plus homologous regions refer to those present on the forward strand in both strains (*i.e*., those that have the same orientation in both the chromosomes), and the plus/minus homologous regions refer to those present on the reverse strand in strain 129Pt in relation to those present on the forward strand in strain 2336 (*i.e*., those that have the opposite orientation, indicative of chromosome inversion). Several large gaps, translocations, and inversions became visible in the alignment generated by NUCmer (Figure 2). Detailed sequence examination revealed that some of these gaps and/or inversions were associated with integrative and conjugative elements.

### Comparison of prophage regions and genomic islands

Prophinder predicted four PRs in strain 2336, but only one in strain 129Pt (Table 2). PR I of strain 2336 was the shortest and had homology to 3 segments totaling ~4,000 bp of bacteriophage HP1 of *H. influenzae *(66-68% nucleotide identity, E-value = 0 to 4e-07). The GC content of all four PRs from strain 2336 (PR I, 40.23%; PR II, 43.95%; PR III, 39.63%; PR IV, 39.85%) was higher than that of the overall genome (37.38% GC). PR II of strain 2336, which had the highest GC content (43.95%) among the four PRs, also contained a 5,679 bp segment (774,075 bp to 779,754 bp) with homology to a region within the genome of *H. influenzae *strain 10810 (72% nucleotide identity, E-value = 0). PR III of strain 2336 was the longest and contained 38 ORFs of unknown function (annotated as encoding hypothetical proteins). The genome of *H. parasuis *strain SH0165 contained several short sequences that had homology to this region (*e.g*., 5 segments totaling ~2,700 bp in the 1,136,975 bp to 1,143,731 bp region, 69-76% nucleotide identity, E-value = 5e-152 to 7e-11). PR IV of strain 2336 was the most conspicuous and contained at least 10 ORFs encoding putative proteins related to bacteriophage structural components. PR IV contained homology to a region of the genome of *H. influenzae *strain 86-028NP (9 segments totaling ~4,700 bp in the 1,707,498 bp to 1,725,687 bp region, 67-84% nucleotide identity, E-value = 3e-126 to 1e-05). Furthermore, the P2 family lysogenic bacteriophage phi-MhaA1-PHL101 from *M. haemolytica *serotype A1 contained some of the ORFs found within PR IV of strain 2336.

**Table 2:** Characteristics of the prophage regions and genomic islands of *H.somni *strains 2336 and 129Pt

Strain	Annotation	Chromosomal location	Size (bp)	GC %	Number of genes
2336	PR I	243787 bp to 255490 bp	11704	40.23	13
	PR II	759194 bp to 783660 bp	24467	43.95	25
	PR III	1001039 bp to 1049028 bp	47990	39.63	55
	PR IV	1301649 bp to 1329985 bp	28337	39.85	39
	GI I	1877815 bp to 1902856 bp	25042	37.00	21
	GI II	1963256 bp to 1992078 bp	28823	42.81	31
	GI III	2124957 bp to 2143800 bp	18844	36.00	18
129Pt	GI I	464154 bp to 472854 bp	8701	37.60	13
	GI II	567352 bp to 574742 bp	7391	33.82	11
	GI III	1524138 bp to 1529684 bp	5547	35.41	7
	PR I	1569138 bp to 1610766 bp	41629	39.37	49
	GI IV	1955573 bp to 1961650 bp	6078	37.86	8
	GI V	1962205 bp to 1966694 bp	4490	31.27	7
	GI VI	1969857 bp to 1974596 bp	4740	38.19	9

**Figure 3:**
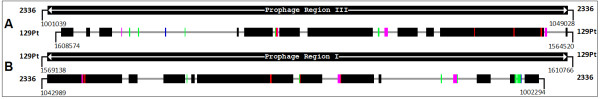
**(A) BLASTN of prophage region III of strain 2336 (47,990 bp) against the chromosome of strain 129Pt and (B) BLASTN of prophage region I of strain 129Pt (41,629 bp) against the chromosome of strain 2336**. The numbers below each of the maps refer to the nucleotide positions in the respective chromosomes. The matches are color-coded according to the BLASTN alignment scores (Red < 40, Blue = 40-50, Green = 50-80, Pink = 80-200, and Black ≥ 200; a gray line indicates the absence of a significant match).

**Figure 4:**
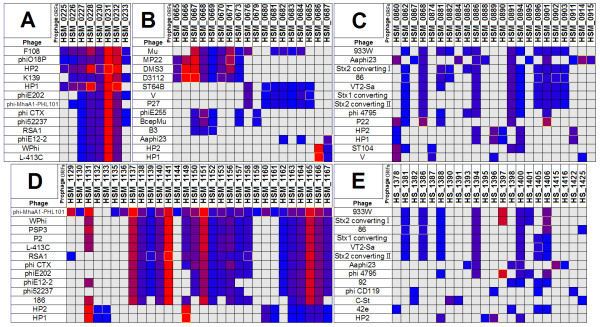
**A matrix view (or heatmap) of homologs of the proteins encoded by the predicted ORFs from prophage regions I-IV of strain 2336 (A-D, respectively) and prophage region I of strain 129Pt (E) within the Prophinder database**. The columns on the top represent the locus tags of the putative coding sequences within the respective prophage regions of the two strains of *H. somni*. The rows on the left represent 13 different bacteriophages within the Profinder database that contain two or more related coding sequences. The matches are color-coded according to the BLASTP E-values (a gradient from red to blue represents E-values from 10e-200 to 0.01 and a gray box indicates the absence of a significant match).

PR I of strain 129Pt was 6,361 bp shorter than PR III of strain 2336, but the two PRs had a similar GC content. A BLASTN analysis indicated that a sequence of ~20,000 bp was conserved between PR I of strain 129Pt and PR III of strain 2336 (96-99% nucleotide identity, E-value = 0). A two-way comparison indicated that an 11,525 bp segment (1,006,964 bp to 1,018,488 bp, 37.38% GC) within PR III, and a 1,255 bp segment from the 5' side of PR III of strain 2336 were absent in strain 129Pt (Figure 3A). A similar comparison indicated that a 5,740 bp segment (1,595,978 bp to 1,601,717 bp, 35.47% GC) within PR I, and a 2,192 bp segment from the 3' side of PR I, of strain 129Pt were absent in strain 2336 (Figure 3B). Bacteriophages within the Prophinder database containing some of the predicted ORFs from PRs I-IV of strain 2336 and PR I of strain 129Pt are shown in Figure 4.

Manual curation indicated that strains 2336 and 129Pt contained 3 and 6 GIs, comprising a total of 72,709 bp and 36,947 bp, respectively (Table 2). GI I of strain 2336 contained 17 ORFs of unknown function and a similar sequence was not found in strain 129Pt or other members of the *Pasteurellaceae*. GI II of strain 2336 was the longest, had a higher GC content (~43%) than the overall genome, and contained 16 ORFs of unknown function. The genome of *P. multocida *strain Pm70 contained several short sequences that had homology to this region (*e.g*., 3 segments totaling 5,433 bp in the 2,174,533 bp to 2,187,234 bp region, 82-99% nucleotide identity, E-value = 0). GI III of strain 2336 contained 4 ORFs encoding putative transposases ([GenBank:HSM_1871, [GenBank:HSM_1874, [GenBank:HSM_1883, and [GenBank:HSM_1887] and 8 ORFs of unknown function. Strain 129Pt lacked an analogous GI, but contained homologs of the genes encoding putative transposases. A 4,640 bp sequence from *Escherichia coli *strain 49 [GenBank:U23723] had homology to GI III of strain 2336 (72% identity across 3,270 bp, E-value = 0).

GI I of strain 129Pt contained an ORF encoding a resolvase/integrase-like protein [GenBank:HS_0445] and 6 ORFs of unknown function. A similar sequence was not found in strain 2336, but *H. parasuis *strain SH0165 contained several short sequences with homology to this region (*e.g*., 3 segments totaling 2,040 bp, 68-79% nucleotide identity, E-values = 0 to 1e-06). GI II of strain 129Pt contained an ORF encoding a putative phage DNA primase-like protein [GenBank:HS_0533] and 9 ORFs of unknown function. A similar sequence was not found in strain 2336 or other members of the *Pasteurellaceae*. GI III of strain 129Pt contained ORFs encoding a putative phage terminase protein [GenBank:HS_1334], a prophage regulatory element [GenBank:HS_1335], an integrase [GenBank:HS_1337], and 4 ORFs of unknown function. A similar sequence was not found in strain 2336, but *Aggregatibacter aphrophilus *strain NJ8700 contained several short sequences that had homology to this region (*e.g*., 2 segments totaling 1,334 bp in the 1,818,930 bp to 1,821,098 bp region, 74-80% nucleotide identity, E-values = 0 to 4e-35). All predicted ORFs from GIs IV and V of strain 129Pt were of unknown function and strain 2336 contained several short sequences that had homology to these regions (*e.g*., 7 segments totaling 2,184 bp in the 1,965,862 bp to 1,990,890 bp region of GI II, 67-92% nucleotide identity, E-values = 9e-42 to 1e-10). The chromosome of strain 2336 had no regions of homology to GI VI of strain 129Pt, but *H. parasuis *strain SH0165 contained several short sequences that had homology to this region (*e.g*., 3 segments totaling 1,573 bp, 68% nucleotide identity, E-values = 2e-51 to 2e-13).

### Comparison of insertion sequences

Insertion sequence finder indicated that strains 2336 and 129Pt contained several IS elements distributed throughout the chromosomes. Not surprisingly, some of these IS elements were found within the PRs and/or GIs described above. Insertion sequence 1016 (IS*1016*), consisting of a transposase [217 amino acids (aa)] flanked by 18-29 bp of terminal inverted repeats, is a member of the IS*1595 *superfamily [35,36]. Strain 2336 contained 4 full-length ([GenBank:HSM_0851], [GenBank:HSM_1211], [GenBank:HSM_1267], and [GenBank:HSM_1883]) and three truncated copies of IS*1016*. GI III of strain 2336 included [GenBank:HSM_1883] and one of the three truncated copies of IS*1016*. Strain 129Pt contained 5 full-length copies of IS*1016 *([GenBank:HS_0324], [GenBank:HS_0642], [GenBank:HS_1116], [GenBank:HS_1679], and [GenBank:HS_1709]). A gene in strain 129Pt (*licA*; [GenBank:HS_1461]), encoding a choline kinase that is necessary for the synthesis of phosphorylcholine [37], appeared to be interrupted due to an insertion-excision event involving IS*1016*, since two partial homologs of the transposase gene occurred upstream of *licA*.

Strain 2336 contained 4 full-length members of the family IS*200*/IS*605 *([GenBank:HSM_0223] was near PR I and [GenBank:HSM_1626] and [GenBank:HSM_1631] were near GI I). Strain 129Pt contained 3 full-length ([GenBank:HS_0486], [GenBank:HS_0583], and [GenBank:HS_1390]) and 6 truncated ([GenBank:HS_0224], [GenBank:HS_0666], [GenBank:HS_0667], [GenBank:HS_0668], [GenBank:HS_0716], and [GenBank:HS_0717]) members of the family IS*200*/IS*605*. PR I of strain 129Pt included [GenBank:HS_1390]. Strain 2336 contained a full-length member of the family IS*30 *([GenBank:HSM_1680]) and a truncated member of the family IS*1182*/IS*5 *([GenBank:HSM_1887]). Strain 2336 contained a full-length member of the family IS*481 *([GenBank:HSM_0451]), whereas strain 129Pt contained a truncated copy of the same ORF ([GenBank:HS_1518]). Strain 2336 also contained truncated members of the family IS*3 *([GenBank:HSM_0531] and [GenBank:HSM_0532]). The closest homologs of *H. somni *IS*200*/IS*605 *and IS*1595 *elements were found in *M*. *haemolytica *(*e.g*., [GenBank:MHA_0340], 92% identity, E-value = 0) and *H*. *influenzae *(*e.g*., [GenBank:CGSHiII_08436], 86% identity, E-value = 0). However, the closest homologs of the IS*30 *element from strain 2336 were found in *A*. *pleuropneumoniae *(*e.g*., [GenBank:APP7_0397], 99% identity, E-value = 0) and *H*. *parasuis *(*e.g*., [GenBank:HAPS_1772], 99% identity, E-value = 8e-180).

### BLAST comparison of protein sets

Strains 2336 and 129Pt contained 1550 predicted protein coding genes in common (bidirectional best hits, at least 90% identity at the predicted protein level). In strain 2336, 440 ORFs could not be assigned a function based on BLAST analysis and were therefore annotated as encoding hypothetical or conserved hypothetical proteins. In strain 129Pt, 429 ORFs were annotated as encoding hypothetical or conserved hypothetical proteins. Among hypothetical proteins that were common to both strains, 30 did not have homologs outside the genus. Pairwise BLAST comparisons indicated that strain 2336 contained 311 putative protein coding genes with no homologs in strain 129Pt (additional file 1). Within this subset, proteins encoded by 302 genes had at least 51 aa and 9 genes ([GenBank:HSM_0528], [GenBank:HSM_0530], [GenBank:HSM_0532], [GenBank:HSM_0603], [GenBank:HSM_1185], [GenBank:HSM_1483], [GenBank:HSM_1636], [GenBank:HSM_1742], and [GenBank:HSM_1743]) had 38-50 aa. Strain 129Pt contained 165 putative protein coding genes with no homologs in strain 2336 (additional file 2). Within this subset, proteins encoded by all 165 genes had at least 51 aa.

In both strains, a vast majority of putative HTGs appeared to have had their origins among members of gammaproteobacteria (mostly within Pasteurellales and Enterobacteriales). Putative HTGs with possible origins among members of betaproteobacteria (27 in strain 2336, 11 in strain 129Pt) and alphaproteobacteria (1 in strain 2336, 6 in strain 129Pt) were also identified. Among HTGs identified were those encoding proteins putatively involved in virulence (*e.g.*, filamentous hemagglutinins, proteases, and antibiotic resistance regulators). A complete list of these genes is available at the 'Organism Details' sections for strains 2336 and 129Pt within IMG. Other strain-specific genes identified encoded DNA methylases (7 in strain 2336, none in strain 129Pt), transposases (8 in strain 2336, 1 in strain 129Pt), ABC transporters (5 in strain 2336, none in strain 129Pt), ATPases (4 in strain 2336, none in strain 129Pt), transcriptional regulators (14 in strain 2336, 3 in strain 129Pt), kinases (2 in strain 2336, 1 in strain 129Pt), and several proteins related to bacteriophage functions (*e.g*., of the 10 integrase/resolvase-related genes found in strain 129Pt, six have no homologs in strain 2336 and of the 7 integrase/resolvase-related genes found in strain 2336, 2 have no homologs in strain 129Pt). Excluding intergenic regions, the total length of sequence that was associated with specific genes in strain 2336 was 254,052 bp (~11% of the genome), and was 98,016 bp (~5% of the genome) in specific genes of strain 129Pt.

### Identification of genes encoding polysaccharide biosynthesis/modification enzymes

A search of the NCBI non-redundant protein database using the BLASTP algorithm identified 17 ORFs that encode putative glycosyltransferases (GTs) in the genomes of strains 2336 and 129Pt. Seven of these ORFs were common to both genomes (at least 96% identity at the predicted protein level), 8 were found only in strain 2336, and 2 were found only in strain 129Pt. Among the ORFs encoding putative GTs common to both strains, 5 contained simple sequence repeats (SSRs), and 4 of the 8 ORFs encoding GTs found in strain 2336 contained SSRs. A list of putative GTs and their SSRs identified in both strains are shown in Table 3. Among the ORFs that encode putative GTs in *H. somni *strains, three ([GenBank:HSM_0148/HS_0275], [GenBank:HSM_0856], and [GenBank:HSM_1552/HS_1067]) had no homologs among other members of the *Pasteurellaceae *and six ([GenBank:HS_0636], [GenBank:HSM_0975], [GenBank:HSM_0977], [GenBank:HSM_0978], [GenBank:HSM_1426], and [GenBank:HSM_1794]) had distant homologs (< 50% identity) among other members of the *Pasteurellaceae *(Table 3). These ORFs were annotated based on their location on the chromosome (*e.g*., the [GenBank:HSM_0975-0979] cluster), comparative analyses using the Swiss-Prot database (*e.g*., [GenBank:HSM_0148], [GenBank:HSM_0856], and [GenBank:HSM_1552]), and their homology to genes encoding putative GTs in non-*Pasteurellaceae *genomes (*e.g*., [GenBank:HS_0636], [GenBank:HSM_1426], and [GenBank:HSM_1794]).

**Table 3:** Putative glycosyltransferase genes of *H.somni *strains

Strain 2336 locus tag, protein,^1^(SSR, if present)_n_	Strain 129Pt locus tag, protein,^1^(SSR, if present)_n_	SWISS-PROT accession number	Closest *Pasteurellaceae *homolog Bacterium, locus tag, protein size,^2^identity	Closest GT homolog (Outside *Pasteurellaceae*) locus tag, protein,^2^identity
[GenBank:HSM_0148], 260 aa, (G)_9_	[GenBank:HS_0275], 259 aa, (G)_6_	[Swiss-Prot:B0UVK9/Q0I1L8]	None	*Acinetobacter johnsonii*, [GenBank:EEY94992], 281 aa, 44%
[GenBank:HSM_0164], **522 aa**, (G)_9_	None	[Swiss-Prot:B0UVM5]	*Actinobacillus succinogenes*, [GenBank:Asuc_0521], 522 aa, 63	*Neisseria mucosa*, [GenBank:EFC88975], 517 aa, 53%
^3^[GenBank:HSM_0398], 346 aa	[GenBank:HS_1612], 346 aa	[Swiss-Prot BOUWU4/Q01570]	*Mannheimia, succiniciproducens *[GenBank:MS2260], 346 aa, 78%	*Yersinia pseudotuberculosis*, [GenBank:YPTB0054], 354 aa, 64%
^3^[GenBank:HSM_0399], 321 aa	[GenBank:HS_1611], 321 aa	[Swiss-Prot B0UWU5/Q0I571]	*Mannheimia succiniciproducens*	*Providencia rettgeri*, [GenBank:EFE51471], [GenBank:MS2259], 321 aa, 67% 322 aa, 63%
^4^[GenBank:HSM_0856], 314 aa	None	[Swiss-Prot:B0UST9]	None	*Rhodopirellula baltica*, [GenBank:RB9243], 326 aa, 35%
^5^[GenBank:HSM_0975a], 157 aa, (CAGT)_18_	^5^[GenBank:HS_0636], 354 aa, (CAGT)_29_	[Swiss-Prot:Q0I2Z7]	*Haemophilus influenzae*, [GenBank:NTHI0365], 312 aa, 38%	*Neisseria meningitidis*, [GenBank:AF355193_3], 311 aa, 35%
[GenBank:HSM_0975], 231 aa, (AGA)_3_	None	[Swiss-Prot:B0UT56]	*Pasteurella dagmatis*, [GenBank:EEX51329], 334 aa, 40%	*Neisseria lactamica*, [GenBank:AAN08512], 251aa, 32%
^6^[GenBank:HSM_0976], 63 aa	[GenBank:HS_0636a]	[Swiss-Prot:B0UT57]	*Pasteurella multocida*, [GenBank:PM1140], 337 aa, 61%	*Streptococcus parasanguinis*, [GenBank:ACF35266], 297 aa, 59%
^7^[GenBank:HSM_0977], 277 aa, (GAGA)_11_	None	[Swiss-Prot:B0UT58]	*Aggregatibacter actinomycetemcomitans *GenBank:D11S_0429], 282 aa, 43%	*Neisseria meningitidis*, [GenBank:NMC1901], 275 aa, 41%
^8^[GenBank:HSM_0978], **271 aa**, (GA)_20_	[GenBank:HS_0637], 158 aa	[Swiss-Prot:B0UT59]	*Aggregatibacter actinomycetemcomitans *[GenBank:D11S_0429], 282 aa, 43%	*Neisseria meningitidis*, [GenBank:CAX50883], 280 aa, 39%
^9^[GenBank:HSM_0979], 293 aa, (CAAT)_33_	[GenBank:HS_0638], 297 aa, (CAAT)_36_	[Swiss-Prot:B0UT60/Q0I2Z5]	*Actinobacillus succinogenes*, [GenBank:Asuc_0526], 265 aa, 52%	*Photobacterium profundum*, [GenBank:PBPRA0217], 253 aa, 41%
[GenBank:HSM_1426], 300 aa	None	[Swiss-Prot:B0UUE7]	*Haemophilus influenzae*, [GenBank:HI0871], 306 aa, 42%	*Fusobacterium nucleatum*, [GenBank:EDK88896], 326 aa, 38%
[GenBank:HSM_1552], 196 aa, (G)_9_	[GenBank:HS_1067], 194 aa, (G)_6_	[Swiss-Prot:B0UUS9/Q0I479]	*None*	*Edwardsiella ictaluri*, [GenBank:NT01EI_2547], 247 aa, 27%
^10^[GenBank:HSM_1794], 311 aa	None	[Swiss-Prot:B0UWB1]	*Mannheimia haemolytica*, [GenBank:MHA_0104], 312 aa, 47%	*Streptococcus agalactiae *[GenBank:AAR29926], 299 aa, 39%
[GenBank:HSM_2001], 242 aa, (GGT)_3_	[GenBank:HS_0116], 242 aa, (GGT)_3_	[Swiss-Prot:B0URS6/Q0I108]	*Aggregatibacter aphrophilus*, [GenBank:NT05HA_1706], 240 aa, 61%	[GenBank:EER56028], 241 aa, 55%
None	[GenBank:HS_0291], 256 aa	[Swiss-Prot:Q0I1K2]	*Mannheimia succiniciproducens *[GenBank:MS0438], 253 aa, 76%	*Vibrio shilonii *[GenBank:EDL53925], 259 aa, 62%
None	[GenBank:HS_0292], 348 aa	[Swiss-Prot:Q0I1K1]	*Actinobacillus *minor NM305, [GenBank:EER47697], 340 aa, 57%	*Vibrio parahaemolyticus*, [GenBank:EED27646], 341 aa, 39%

^1^Simple sequence repeats identified within the predicted ORF.

^2^Identity at the predicted protein level as compared to strain 2336 or strain 129Pt full-length or partial homolog.

^3^HSM_0398 and 0399 are predicted to encode lipopolysaccharide heptosyltransferases II and I, respectively.

^4^HSM_0856 contains 28 copies of CAGT 67 bp upstream of the predicted start codon.

^5^HS_0636 (*lob2D*), encoding a putative glycosyl transferase [13], is truncated in strain 2336 (HSM_0975a).

^6^HSM_0976 (*lob2C*), encoding a putative glycosyltransferase-like protein, has an 117 bp homolog in strain 129Pt (HS_0636a).

^7^HSM_0977 (*lob2B*) has been predicted to encode an *N*-acetylglucosamine transferase [5].

^8^HSM_0978 (*lob2A*), encoding a putative glycosyl transferase, had 66% identity to HSM_0977 (*lob2B*) at the predicted protein level.

^9^HSM_0979/HS_0638 (*lob1*) has been predicted to encode a galactosyl transferase [7].

^10^HSM_1794 contains 4 copies of CAAGCCTACAAGCCTACAAGCCTA 262 bp upstream of the predicted start codon.

The lipooligosaccharide biosynthesis (*lob*) gene cluster consisting of *lob1 *and *lob2ABCD *ORFs encoding glycosyltransferases involved in attaching the outer core glycoses of the LOS was previously identified in strain 738, which is an LOS phase variant of strain 2336 [5,7]. Strain 129Pt encoded full-length homologs of *lob1 *and *lob2D*, but only the 5' ends of *lob2A *and *lob2C *and lacked *lob2B *[13]. Strain 2336 contained full-length homologs of *lob1 *and *lob2ABC*, but a truncated homolog of *lob2D *(table 3). The variations in the *lob *loci in the genomes of strains 2336 and 129Pt correlate with the differences in the structures of the LOS of these strains, as determined by NMR spectroscopy and mass spectrometry [16,38]. In addition, both strains contained ORFs encoding a phosphoheptose isomerase ([GenBank:HSM_0840] and [GenBank:HS_1238], *gmhA*), D,D-heptose 1,7-bisphosphate phosphatase ([GenBank:HSM_0572] and [GenBank:HS_1532], *gmhB*), ADP-L-glycero-*D*-mannoheptose-6-epimerase ([GenBank:HSM_0397] and [GenBank:HS_1613], *rfaD*), bifunctional heptose 7-phosphate kinase/heptose 1-phosphate adenyltransferase ([GenBank:HSM_0925] and [GenBank:HS_0576, *rfaE*), and bifunctional *N*-acetylglucosamine-1-phosphate uridyltransferase/glucosamine-1-phosphate acetyltransferase ([GenBank:HSM_0204] and [GenBank:HS_0333], *glmU*). These five genes/enzymes were predicted to be involved in LOS core biosynthesis in strains 2336 and 129Pt.

Strain 2336 also contained a locus that encodes proteins putatively involved in exopolysaccharide (EPS) and/or LOS biosynthesis ([GenBank:HSM_1061] or *csrA*, carbon storage regulator; [GenBank:HSM_1062] or *manB *phosphomannomutase; [GenBank:HSM_1063] or *galU*, UTP-glucose-1-phosphate uridylyltransferase). Strain 129Pt contained a similar locus ([GenBank:HS_1117] to [GenBank:HS_1119]) that had an IS*1016 *element ([GenBank:HS_1116], transposase) adjacent to it. Furthermore, both strains contained ORFs encoding a putative UDP-glucose 4-epimerase ([GenBank:HSM_1256] and [GenBank:HS_0789], *galE*), phosphoglucomutase ([GenBank:HSM_1832] and [GenBank:HS_1670], *pgmB*), and dTDP-glucose 4,6-dehydratase ([GenBank:HSM_1118] and [GenBank:HS_0707], *rmlB*). These three genes/enzymes were predicted to be involved in polysaccharide biosynthesis/modification.

### Acquisition and loss of genes encoding filamentous hemagglutinins

**Figure 5:**
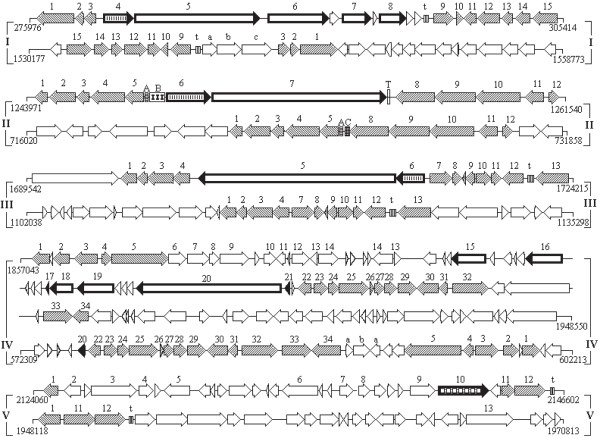
**Comparison of the chromosomal regions of strains 2336 and 129Pt that encode putative filamentous hemagglutinins and/or subtilisin**. The numbers on either side of the maps refer to the nucleotide positions in the respective chromosomes. Although the loci are of different sizes, each map within a locus is drawn to scale. Bold arrows represent genes of interest. Arrows with identical slashes inside them represent orthologous genes among the two strains. Unmarked white arrows represent strain-specific genes, genes found at a different location in the other chromosome, or genes encoding hypothetical proteins. Within each locus, the top portion represents strain 2336 and the bottom portion represents strain 129Pt. Locus I. 1. Hypothetical protein ([GenBank:HSM_0264]), 2. Methylglyoxal synthase, 3. Hypothetical protein ([GenBank:HSM_0266]), 4. Hemolysin activation/secretion protein (*fhaC*), 5, 6, 7, 8. Filamentous hemagglutinin outer membrane protein (*fhaB*), t. tRNA, 9. Cystathionine gamma-synthase, 10. Thioredoxin, 11. Chromosomal replication initiator DnaA, 12. Uracil-xanthine permease, 13. Uracil phosphoribosyltransferase, 14. Glutamine amidotransferase class-II, 15. Glutamate dehydrogenase, a,b,c. Chromosome/plasmid partition locus. Locus II. 1. DNA-binding transcriptional activator GutM, 2. Sorbitol-6-phosphate dehydrogenase, 3. PTS system glucitol/sorbitol-specific IIA component, 4. PTS system, glucitol/sorbitol-specific, IIBC subunit, 5. PTS system, glucitol/sorbitol-specific, IIC subunit, A. 153 bp sequence found only in strains 129Pt and 2336, B. 500 bp sequence with homology to type III restriction-modification genes, C. 173 bp sequence found only in strain 129Pt, 6-*fhaC*, 7. *fhaB*, T. Transposon-related sequence, 8. UbiH/UbiF/VisC/COQ6 family ubiquinone biosynthesis hydroxylase, 9. Polyprenyl-6-methoxyphenol 4-hydroxylase, 10. Peptidase M24, 11. YecA family protein, 12. Cell division protein ZapA. Locus III. 1. Biotin synthase, 2. Thiamine ABC transporter, ATP-binding protein, 3. Thiamine ABC transporter, inner membrane subunit, 4. Thiamine ABC transporter, periplasmic binding protein, 5. *fhaB*, 6. *fhaC*, 7. Na^+ ^antiporter NhaC, 8. Predicted primosomal replication protein N, 9. ECF subfamily RNA polymerase sigma-24 factor, 10. Oxidoreductase molybdopterin binding, 11. Ferric reductase domain-containing protein, 12. Permease, t. tRNA, 13. Ribosomal RNA large subunit methyltransferase L. Locus IV. 1. O-succinylbenzoate synthase, 2. Naphthoate synthase, 3. Phosphopyruvate hydratase, 4. Type I restriction enzyme, specificity subunit, 5. HsdR family type I site-specific deoxyribonuclease, 6. Abi family protein, 7. Restriction-modification system DNA specificity subunit, 8. Filamentation induced by cAMP protein Fic, 9. Type I restriction-modification system M subunit, 10. Phage integrase family protein, 11. Restriction-modification system DNA specificity subunit, 12. Acyltransferase, 13. Transposase, 14. Transposase, 15, 16, 17, 18, 19, 20. *fhaB*, 21. *fhaC*, 22. Hypothetical protein, 23. Ubiquinone/menaquinone biosynthesis methyltransferase, 24. Hypothetical protein, 25. Probable ubiquinone biosynthesis protein ubiB, 26, 27, 28. Twin-arginine translocation proteins, 29. Delta-aminolevulinic acid dehydratase, 30. Phosphoribosylglycinamide formyltransferase, 31. S-ribosylhomocysteine lyase (*luxS*), 32. Transketolase, 33. CTP synthase, 34. Acetyltransferase, a. Restriction-modification system DNA specificity subunit, b. Phage integrase. Locus V. 1. LysR family transcriptional regulator, 2. Phage repressor, 3. Transposase, 4. Phage transposase, 5. Transposase, 6. DNA methylase, 7. Transposase, 8. Transposase, 9. AAA ATPase, 10. Peptidase S8 and S53 subtilisin kexin sedolisin, 11. Permease DsdX, 12. D-serine dehydratase, 13. DNA topoisomerase III, t. tRNA.

The chromosome of *H. somni *strain 2336 contained four loci that have twelve putative genes encoding proteins homologous to FhaB, and four putative genes encoding proteins homologous to FhaC. Locus I of strain 2336 was 17,222 bp (GC content of 40%) and contained genes encoding four FhaB homologs ([GenBank:HSM_0268], [GenBank:HSM_0270], [GenBank:HSM_0272], and [GenBank:HSM_0274]) and one FhaC homolog ([GenBank:HSM_0267]). No transposon or phage sequences were present in this locus. In contrast, strain 129Pt had a locus containing genes that flanked locus I of strain 2336, but did not contain the FhaB and FhaC homologs (Figure 5, locus I). Locus II of strain 2336 was 6,712 bp (GC content of 26%) and contained genes encoding FhaB ([GenBank:HSM_1090]) and FhaC ([GenBank:HSM_1089]), which appeared to be associated with a transposon. Strain 129Pt had a locus containing genes that flanked locus II of strain 2336, but did not contain the FhaB and FhaC homologs (Figure 5, locus II).

Locus III of strain 2336 was 14,066 bp (GC content of 37%) and contained genes encoding FhaB ([GenBank:HSM_1489]) and FhaC ([GenBank:HSM_1490]). The *fhaB *(12,288 bp) of this locus was the second largest gene in the genome and the largest among the 12 homologs. This gene encoded a putative protein homologous to the high molecular weight immunoglobulin-binding protein of *H. somni *and the large supernatant proteins (Lsp1 and Lsp2) of *H*. *ducreyi *that have been previously described [39]. No transposon or phage regions were apparent in this locus. Strain 129Pt had a locus containing genes that flank locus III of strain 2336, but did not contain the FhaB and FhaC homologs (Figure 5, locus III). Locus IV of strain 2336 was 21,841 bp (GC content of 38%) and contained genes encoding six FhaB homologs ([GenBank:HSM_1638], [GenBank:HSM_1641], [GenBank:HSM_1643], [GenBank:HSM_1646], [GenBank:HSM_1647], [GenBank:HSM_1651]). A truncated gene encoding FhaC ([GenBank:HSM_1653], 98 aa) was also found in this locus. A partial homolog of [GenBank:HSM_1647] was found in strain 129Pt ([GenBank:HS_0540], 153 aa), which appeared to be the only region in the chromosome of strain 129Pt with a sequence related to the *fhaB *genes (Figure 5, locus IV).

**Figure 6:**
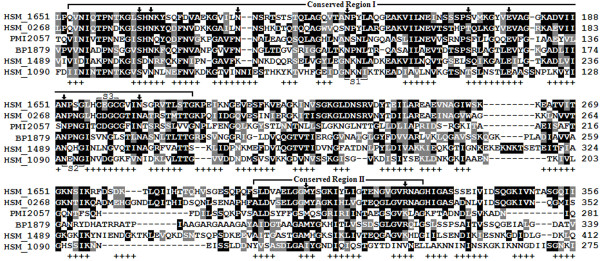
**ClustalW-BOXSHADE multiple sequence alignment of N-terminal fragments of FhaB homologs from strain 2336 and comparison to HpmA of *P*. *mirabilis *and FHA of *B*. *pertussis***. GenBank accession numbers of protein sequences are as follows: [GenBank:HSM_1651] (*fhaB*) filamentous hemagglutinin outer membrane protein [GenBank:ACA31420]; [GenBank:HSM_0268], (*fhaB*) filamentous hemagglutinin outer membrane protein [GenBank:ACA31896]; [GenBank:PMI2057] (*hpmA*) hemolysin [GenBank:CAR44120]; [GenBank:BP1879] (*fhaB*) filamentous hemagglutinin/adhesin, [GenBank:CAE42162]; [GenBank:HSM_1489] (*fhaB*) cysteine protease domain-containing protein [GenBank:ACA31239]; and [GenBank:HSM_1090] (*fhaB*) filamentous hemagglutinin outer membrane protein [GenBank:ACA30807]. Homologous regions are box-shaded black (identical amino acid residues) and gray (conserved amino acid substitutions). Arrows point to arginine, asparagine, glutamic acid, methionine, and serine residues that are shown to be involved in FHA secretion. Regions that correspond to the secondary structural components of FHA and HpmA are marked with a plus sign. S1 (*NXXL*), S2 (*NXXG*), and S3 (*CXXC*) indicate motifs that may be involved in stabilization of the helical structure of FHA and/or HpmA. Numbers at the end of each sequence denote amino acid positions.

The twelve putative FhaB homologs found in the four loci of strain 2336 varied in size, with the smallest and largest being 83 aa and 4,095 aa, respectively. Phylogenetic comparison indicated that the four FhaB homologs within locus I were most closely related to each other as were the six FhaB homologs within locus IV (data not shown). The FhaC homologs of loci I (581 aa) and III (586 aa) were more closely related to each other than to FhaC from locus II (450 aa). Multiple sequence alignment of N-terminal fragments of FhaB homologs from the four loci of strain 2336 with those of *Bordetella pertussis *FHA and *Proteus mirabilis *HpmA showed that they contain several common features (Figure 6). Of the many residues that are shown to be involved in *B*. *pertussis *FHA secretion, 6 (four asparagine and one each of serine and glutamic acid) were conserved in all six homologs and 2 (an asparagine and a methionine) were conserved in five of the homologs (Figure 6). However, the *NPNG *(Figure 6, S2) and *CXXC *(Figure 6, S3) motifs that may play a role in stabilization of the helical structure were conserved in only three homologs.

### Acquisition of a gene encoding subtilisin-like protease

*H. somni *strain 2336 GI III contained a gene encoding a putative subtilisin-like serine protease ([GenBank:HSM_1889], 730 aa; Figure 5, locus V, arrow (gene) 10). A BLASTP search revealed that the non-redundant GenBank database contained several proteins that were homologous to [GenBank:HSM_1889], with the closest relative being an *E. coli *protein ([GenBank:AAA64865], 75% identity, E-value = 0). The NCBI protein clusters database lists [GenBank:HSM_1889] as one of the 16 members of the [GenBank:CLSK923564] cluster (peptidase S8 and S53, subtilisin, kexin, sedolisin). Among the proteins of this cluster, homologs from *Ralstonia eutropha *JMP134 ([GenBank:Reut_C6419], 45% identity, E-value = 1e-178), *Pseudomonas fluorescens *Pf0-1 ([GenBank:Pfl01_5697], 35% identity, E-value = 2e-116), and *Pseudomonas syringae *pv. phaseolicola 1448A ([GenBank:PSPPH_0180], 35% identity, E-value = 1e-115) are listed in the prokaryotic subtilase database as subtilases of the D-H-S family.

**Figure 7:**
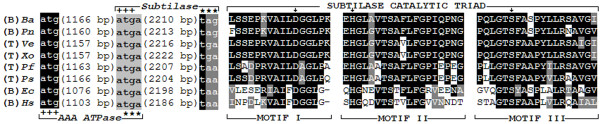
**Comparison of 8 members of the NCBI protein cluster CLSK923564**. *Ba*; *Burkholderia ambifaria *AMMD [GenBank:YP_773860], *Pn*; *Polaromonas naphthalenivorans *CJ2 [GenBank:YP_982671], *Ve*; *Verminephrobacter eiseniae *EF01-2 [GenBank:YP_998470], *Xo*; *Xanthomonas oryzae *pv. oryzae PXO99A [GenBank:YP_001913386], *Pf*; *P*. *fluorescens *Pf0-1 [GenBank:YP_351424], *Ps*; *P*. *syringae *pv. phaseolicola 1448A [GenBank:YP_272486], *Ec*; *E*. *coli *O127:H6 str. E2348/69 [GenBank:YP_002329331], *Hs*; *H. somni *strain 2336 [GenBank:YP_001785203]. Gene features are shown on the left side: Start and stop codons of ORFs encoding ATPase and sutbilase are marked with plus and asterisk signs, respectively. The length of each ORF is measured from the first base after the start codon to the last base before the stop codon. The 4 bp gene overlap is box-shaded light gray. (B) denotes genes found within prophage-like sequences and (T) denotes genes associated with transposons. ClustalW-BOXSHADE multiple sequence alignment of subtilase motifs containing the catalytic triad is shown on the right side: Homologous regions are box-shaded black (identical amino acid residues) and gray (conserved amino acid substitutions). Arrows point to the conserved residues Asp-His-Ser of subtilases of the D-H-S family.

Genomic comparison of members of the [GenBank:CLSK923564] cluster revealed that in 3 cases (*Arthrobacter aurescens *TC1, *R. eutropha *JMP134, and *Xanthomonas campestris *pv. *vesicatoria *str. 85-10), the ORF encoding subtilisin was found on plasmids. In the case of *Delftia acidovorans *SPH-1, *Burkholderia ambifaria *AMMD, *Polaromonas naphthalenivorans *CJ2, *Chelativorans *sp. BNC1, and *E. coli *O127:H6 str. E2348/69, the ORF encoding subtilisin was found within a prophage region. Furthermore, in the case of *Photorhabdus luminescens *subsp. *laumondii *TTO1, *P. syringae *pv. phaseolicola 1448A, *P. fluorescens *Pf0-1, *Verminephrobacter eiseniae *EF01-2, and *Xanthomonas oryzae *pv. oryzae PXO99A, the ORF encoding subtilisin appeared to be associated with genes encoding transposases. However, in *Chromohalobacter salexigens *DSM 3043 and *Anaeromyxobacter *sp. K, the ORF encoding subtilisin was not associated with prophage or transposase sequences. Interestingly, in 15 host species the subtilisin ORF formed an operon with an ORF encoding homologous ATPases of the AAA family. In the case of *A. aurescens *TC1, a transposon insertion appears to have disrupted the AAA ATPase-subtilisin operon. The two ORFs have a 4 bp overlap in 8 species and appear to be co-transcribed (Figure 7, left side). Furthermore, comparison of subtilisin sequences from these 8 species indicated that motifs containing the catalytic triad (Asp-His-Ser) were conserved (Figure 7, right side).

### Comparison of genes encoding transferrin-binding proteins

A comparison of the genomes of *H. somni *strains 2336 and 129Pt revealed that both strains contained genes encoding TbpA ([GenBank:HS_0449] and [GenBank:HSM_0750]) and TbpB ([GenBank:HS_0448] and [GenBank:HSM_0749]). In strain 129Pt, GI I was identified immediately upstream of the *tbp *locus. Furthermore, homologs of *H. somni *strain 649 TbpA2 were present in strain 129Pt ([GenBank:HS_0582], heme uptake protein) and strain 2336 ([GenBank:HSM_0931], TonB-dependent receptor; [GenBank:HSM_0932], TonB-dependent receptor plug; and [GenBank:HSM_1988], TonB-dependent lactoferrin and transferrin receptor). Pairwise BLAST analysis identified several other putative genes encoding proteins that may also be involved in iron transport. In strain 129Pt these included [GenBank:HS_0069], iron-regulated outer membrane protein; [GenBank:HS_0181], TonB-dependent outer membrane receptor; [GenBank:HS_0728], hemin receptor outer membrane protein; and [GenBank:HS_1306], iron-regulated outer membrane protein. In strain 2336 they included [GenBank:HSM_0047], TonB-dependent receptor; [GenBank:HSM_1168], TonB-dependent hemoglobin/transferrin/lactoferrin family receptor; [GenBank:HSM_1176], outer membrane hemin receptor protein, and [GenBank:HSM_1962], TonB-dependent receptor. Two of the genes, [GenBank:HS_1306] and [GenBank:HSM_1168], were strain-specific. Among others, HS_0582 was associated with a transposase ([GenBank:HS_0583]) and [GenBank:HSM_1168] was found near PR IV.

### Overabundance of genes encoding adhesin-like proteins

Strain 129Pt contained sixteen genes randomly distributed throughout the chromosome ([GenBank:HS_0209], [GenBank:HS_0383], [GenBank:HS_0478], [GenBank:HS_0589], [GenBank:HS_0602], [GenBank:HS_0790], [GenBank:HS_1058], [GenBank:HS_1085], [GenBank:HS_1154], [GenBank:HS_1185], [GenBank:HS_1234], [GenBank:HS_1512], [GenBank:HS_1543], [GenBank:HS_1563], [GenBank:HS_1616], and [GenBank:HS_1632]) with homology to genes that encode proteins of the *Yersinia *adhesin (YadA) superfamily [13]. Strain 2336 also contained sixteen genes randomly distributed throughout the chromosome ([GenBank:HSM_0077], [GenBank:HSM_0338], [GenBank:HSM_0346], [GenBank:HSM_0377], [GenBank:HSM_0394], [GenBank:HSM_0708], [GenBank:HSM_0844], [GenBank:HSM_0938], [GenBank:HSM_0953], [GenBank:HSM_1022], [GenBank:HSM_1212], [GenBank:HSM_1257], [GenBank:HSM_1484], [GenBank:HSM_1542], [GenBank:HSM_1571], [GenBank:HSM_1793]) with homology to genes that encode proteins of the YadA superfamily. Adhesin-encoding genes [GenBank:HS_0209] (15,431 bp) and [GenBank:HSM_1257] (13,970 bp) were the largest among all protein coding genes predicted in the chromosomes of strains 129Pt and 2336, respectively. Within this gene repertoire, [GenBank:HSM_0938] (388 aa) and [GenBank:HS_0589] (386 aa) were 85% similar to each other at the predicted protein level (75% identity, Score = 535 bits, E-value = 1e-156) and were associated with genes encoding putative ABC transporters ([GenBank:HSM_0939-0943] and [GenBank:HS_0590-0594], respectively, 99% identity, E-value = 0 to 5e-151). In addition, [GenBank:HSM_0077] (4063 aa) and [GenBank:HS_0209] (5143 aa) were 65% similar to each other at the predicted protein level (53% identity, Score = 1009 bits, E-value = 0) and were also associated with genes encoding putative ABC transporters ([GenBank:HSM_0079-0081] and [GenBank:HS_0210-0212], respectively, 99% identity, E-value = 2e-146 to 3e-132). Furthermore, although homologs of the *H. influenzae *fimbrial gene cluster (*hifABCDE*) were absent in both strains of *H. somni*, homologs of type IV pili genes (*pilABCD*) occurred in strains 2336 and 129Pt (*e.g*., [GenBank:HSM_0123] and [GenBank:HS_0250], [GenBank:HSM_0217] and [GenBank:HS_1430, [GenBank:HSM_0755 and [GenBank:HS_0457], and [GenBank:HSM_0756] and [GenBank:HS_0458], 40-69% identity, E-values = 1e-168 to 1e-38). In addition, both strains contained a gene that encoded a putative pseudopilin (*pulG*, [GenBank:HS_0264] and [GenBank:HSM_0137], 54% identity to NTHI1109, E-value = 9e-51) that may facilitate type II secretion.

### Identification of genes encoding transcriptional regulators and drug/metal resistance

Pairwise BLAST comparisons indicated that strain 2336 contained 14 genes encoding transcriptional regulators with no homologs in strain 129Pt. These included two regulators each that belong to the TetR ([GenBank:HSM_1191] and [GenBank:HSM_1734]) and MerR ([GenBank:HSM_1728] and [GenBank:HSM_1741]) families, one each that belonged to the LysR ([GenBank:HSM_0806]), OmpR ([GenBank:HSM_0817]), and MarR ([GenBank:HSM_1737]) families, and a member of an unassigned family ([GenBank:HSM_0489]; [GenBank:COG2865K]). Among these, members of the TetR, MerR, and MarR families were found within GI II. Strain 2336 also contained a gene adjacent to *tetR *encoding a tetracycline resistance antiporter (*tetH*; [GenBank:HSM_1735]). The NCBI protein clusters database lists [GenBank:HSM_1734] as a member of the PRK13756 cluster (TetR; tetracycline repressor of *Leuconostoc citreum*[GenBank:COG1309K]). The closest homologs of [GenBank:HSM_1734] and [GenBank:HSM_1735] were found in *P. multocida *(*e.g*., [GenBank:AAC43249] and [GenBank:AAC43250] encoding a repressor protein and a tetracycline resistance protein, respectively, 99% identity, E-values = 0 to 1e-116). In addition to [GenBank:HSM_1737], strain 2336 also contained [GenBank:HSM_1736] (encoding small multidrug resistance protein). [GenBank:HSM_1191] is listed as one of the members of the [GenBank:CLSK391246] cluster (TetR family transcriptional regulator). This regulator gene was associated with two genes encoding an ABC-type transport system ([GenBank:HSM_1192] and [GenBank:HSM_1193]). The closest homologs of [GenBank:HSM_1191] were found among members of Firmicutes, Spirochaetes, and Fusobacteria (*e.g*., [GenBank:EFM38698] encoding a putative TetR family transcriptional regulator in *Eubacterium yurii *subsp. *margaretiae*, 99% identity, E-value = 1e-104), but not the *Pasteurellaceae*. The two MerR homologs of strain 2336 had only 39% identity to each other (E-value = 7e-23) and both were conserved in members of the *Pasteurellaceae *(*e.g*., [GenBank:HAPS_1832] from *H*. *parasuis *and [GenBank:PM1941] from *P. multocida*, 85-99%% identity, E-values = 4e-57 to 4e-70). However, the MerR homologs have been included in different protein clusters ([GenBank:HSM_1728] in [GenBank:CLSK892364] and [GenBank:HSM_1741] in [GenBank:CLSK2299246]) and appear to regulate different functions. Since [GenBank:HSM_1728] was associated with genes involved in copper metabolism ([GenBank:HSM_1729] encodes a metal binding protein, [GenBank:HSM_1730] encodes a multicopper oxidase, and [GenBank:HSM_1731] encodes a copper-translocating ATPase), it may encode a copper efflux regulator, CueR. However, [GenBank:HSM_1741] may be involved in zinc homeostasis since it was associated with zinc-responsive genes ([GenBank:HSM_1740] encodes a zinc efflux protein and [GenBank:HSM_1739] encodes a zinc-dependent hydrolase). The NCBI protein clusters database lists [GenBank:HSM_0806] as one of the members of the [GenBank:CLSK797597] cluster (LysR family transcriptional regulator, [GenBank:COG0583K]). The closest homologs of [GenBank:HSM_0806] were found in *Pasteurella dagmatis *([GenBank:EEX49428], 65% identity, E-value = 5e-113), *H. influenzae *([GenBank:NTHI0936], 59% identity, E-value = 1e-101), and several species of Streptococci (*e.g*., [GenBank:SUB1393] and [GenBank:SPy_1634], 50-52% identity, E-values = 2e-86 to 1e-83). In strain 2336, [GenBank:HSM_0817] encoded a putative OmpR family response regulator whereas [GenBank:HSM_1124] encoded a putative ArcA family response regulator. These response regulators have very low homology to each other (33% identity, E-value = 7e-34). However, their amino-termini contained two conserved aspartic acid residues (Asp-11 and Asp-56) that may serve as phosphate acceptors and their carboxy-termini contained several conserved residues that may be involved in DNA binding (data not shown). Strains 2336 and 129Pt also contained genes that encoded putative histidine kinases ([GenBank:HSM_0483] and [GenBank:HS_1510], [GenBank:HSM_0727] and [GenBank:HS_0402], and [GenBank:HSM_1378 and [GenBank:HS_0900]). Whereas [GenBank:HSM_1378] and [GenBank:HS_0900] had an ORF downstream that encodes a putative cognate response regulator ([GenBank:HSM_1379] and [GenBank:HS_0901]), [GenBank:HSM_0727] and [GenBank:HS_0402] had an ORF downstream that encodes a transcriptional regulator with an N-terminal XRE-type helix-turn-helix domain ([GenBank:HSM_0728] and [GenBank:HS_0403]). Strain 2336 contained an additional gene that encoded a putative histidine kinase ([GenBank:HSM_0824]) that had a homolog in *P. multocida *strain Pm70 ([GenBank:PM1380], 58% identity, E-value = 0), but not in strain 129Pt.

### Identification of genes encoding restriction-modification enzymes

Type I and Type II restriction-modification (RM) systems comprise the most frequently encountered DNA modifying enzymes among eubacteria. Strain 2336 contained ORFs that putatively encode proteins of the Type I RM system ([GenBank:HSM_1615], *hsdR*, site-specific deoxyribonuclease; [GenBank:HSM_1617], *hsdS*, DNA specificity subunit; [GenBank:HSM_1619], *hsdM*, DNA methylation subunit). Although strain 129Pt lacked *hsdM*, it contained a full-length *hsdR *([GenBank:HS_0559]) and two truncated *hsdS *([GenBank:HS_0554] and [GenBank:HS_0556]) orthologs. Strain 2336 also contained an operon putatively encoding enzymes of the Type II RM system ([GenBank:HSM_0801], *M.hsoI*, DNA-cytosine methyltransferase; [GenBank:HSM_0802], *R.hsoI*, restriction endonuclease). At the predicted protein level, M.HsoI has 68% identity to M.HinP1I ([GenBank:AAW33810], E-value = 2e-121) and R.HsoI has 72% identity to R.HinP1I ([GenBank:AAW33811], E-value = 3e-105) of *H. influenzae *P1. Strain 129Pt lacked *M.hsoI *and *R.hsoI *but contained an operon putatively encoding a *Bcg*I-like RM system ([GenBank:HS_0430], site-specific DNA-methyltransferase; [GenBank:HS_0431], restriction enzyme; [GenBank:HS_0432], restriction enzyme; [GenBank:HS_0433], hypothetical protein) that is absent in strain 2336.

### Identification of other genes relevant to metabolism and survival

Both *H. somni *strains contained ORFs encoding proteins putatively involved in the transport and metabolism of fucose ([GenBank:HSM_0580] and [GenBank:HS_1451] to [GenBank:HSM_0585] and [GenBank:HS_1446]), mannose ([GenBank:HSM_0956] and [GenBank:HS_0605] to [GenBank:HSM_0960] and [GenBank:HS_0609]), xylose ([GenBank:HSM_0933] and[GenBank:HS_0584] to [GenBank:HSM_0937] and [GenBank:HS_0588]), galactitol ([GenBank:HSM_1030] and [GenBank:HS_1146] to [GenBank:HSM_1036] and [GenBank:/HS_1140]), mannitol ([GenBank:HSM_0825] and [GenBank:HS_1252] to [GenBank:HSM_0827] and [GenBank:HS_1250]), and *myo*-inositol ([GenBank:HSM_0425] and [GenBank:HS_1586] to [GenBank:HSM_0435] and [GenBank:HS_1576]). Furthermore, strain 2336 contained a locus with three ORFs encoding proteins putatively involved in galactose utilization (*galTKM*; [GenBank:HSM_0108] to [GenBank:HSM_0110]), whereas strain 129Pt contained only *galM *([GenBank:HS_0236]) and truncated *galK *([GenBank:HS_0235]) orthologs. However, both strains have *galE*, and strain 129Pt has galactose in its LOS (38), indicating that it can metabolize galactose.

Strain 2336-specific genes encoding proteins putatively involved in carbohydrate metabolism included [GenBank:HSM_0818] (ribokinase-like domain-containing protein), [GenBank:HSM_0819] (ketose-bisphosphate aldolase), [GenBank:HSM_0820] (sugar-related regulatory protein), [GenBank:HSM_0821] (sugar binding protein), [GenBank:HSM_0822] (monosaccharide-transporting ATPase), and [GenBank:HSM_0823] (ABC type sugar transporter). Strain 2336 also contained ORFs encoding a cyclase family protein and a membrane-spanning protein ([GenBank:HSM_1907] and [GenBank:HSM_1908]). These ORFs appeared to form an operon with ORFs encoding proteins putatively involved in butyrate/pyruvate metabolism ([GenBank:HSM_1903] to [GenBank:HSM_1906]). Furthermore, strain 2336 contained an ORF that encoded an oligopeptide permease ABC transporter homolog ([GenBank:HSM_0695]). The putative Opp protein of strain 2336 was related to OppB proteins involved in peptide transport in *E. coli *and *Bacillus subtilis *(*e.g*., [GenBank:AAC74326] and [GenBank:OPPB_BACSU], respectively, 46-76%% identity, E-values = 2e-138 to 1e-72).

GI I of strain 129Pt contained a gene encoding a putative phosphotransferase system protein ([GenBank:HS_0437], cellobiose-specific IIC component) whose homologs are found in *M*. *haemolytica *([GenBank:MHA_2539], 94% identity, E-value = 0) and *H*. *parasuis *([GenBank:HAPS_1535], 86% identity, E-value = 0). Strain 129Pt also contained a gene encoding a virulence-associated protein E ([GenBank:HS_0427]). This protein was related to the VirE proteins encoded by genes found on *Staphylococcus phage phi2958PVL *([GenBank:BAG74398], 31% identity, E-value = 4e-36) and *Enterococcus phage phiFL4A *([GenBank:ACZ64175], 33% identity, E-value = 3e-31). Strain 129Pt contained genes ([GenBank:HS_0631] and [GenBank:0632]) encoding a putative serine/threonine protein kinase-phosphatase pair. Strain 129Pt also contained loci encoding proteins putatively involved in thiamine biosynthesis (*thiMDE*; [GenBank:HS_0090-0092]), tellurite resistance (*terDEZ*; [GenBank:HS_0633-0635]), and lysine degradation (*cadBA*; [GenBank:HS_1006 and [GenBank:HS_1007]).

## Discussion

Genetic events such as deletions, duplications, insertions, and inversions are relatively common in bacterial chromosomes as a result of bacteriophage infection, integration and excision of plasmids, transpositions, and/or replication-mediated translocations [40,41]. In addition, different prophages embedded within a single chromosome can contain similar genes encoding integration and structural functions, and it is not uncommon for these genes to undergo homologous recombination. One of the consequences of such homologous recombination is the rearrangement of the host chromosome [42]. These events are known to be the precursors of evolution and can bring about a significant change in the number, linear order, and orientation of genes on the circular chromosomes of different strains/species of closely related bacteria [43].

The presence of PRs in the chromosomes of strains 2336 and 129Pt was a notable feature since the number and diversity of genes associated with these PRs far exceeded those described in *H. influenzae *strains Rd KW20 and 86-028NP [44,45]. The difference in the size of the chromosomes of strains 2336 and 129Pt was partly due to PRs and associated genes. Similar observations have been made in other bacteria wherein prophage-associated sequences constitute a large portion of strain-specific DNA [42]. Although one of the functions of RM systems is to afford protection against bacteriophage attack (the "*cellular defense hypothesis*"), it is interesting to note that both strains contain several prophage-like sequences despite the presence of genes encoding putative RM systems in their chromosomes. The lack of ORFs encoding HsdM, M.HsoI, and R.HsoI in strain 129Pt indicates that these systems are not absolutely essential for cell survival. Their absence may also partially explain the relative ease with which this strain can be transformed in the laboratory.

Biosynthesis of polysaccharides requires a multitude of GTs, which catalyze the transfer of sugars from an activated donor to an acceptor molecule and are usually specific for the glycosidic linkages created [46]. Intra and interspecies divergence of genes encoding GTs are not uncommon. Phase-variable LOS is an important virulence factor of pathogenic strains of *H. somni*. Phase-variation of *H. somni *LOS has been shown to be due to the presence of SSRs in genes that encode GTs and enzymes involved in assembling non-glycose LOS components such as phosphorylcholine [5,7,37]. The genes *lob1, lob2AB*, and *lob2D *contain SSRs either just before the start codons or within the open reading frame [47]. In addition, [GenBank:HSM_0148], [GenBank:HSM_0164], [GenBank:HSM_0975], and [GenBank:HSM_1552] also contain SSRs that may be responsible for LOS phase variation, but require additional experimental investigation. Most *H. somni *strains also produce a biofilm-associated EPS consisting primarily of mannose and galactose [47]. Although characterization of some of the genes involved in the biosynthesis and/or modification of *H. somni *LOS/EPS has been determined, the identification of several more has been facilitated by comparative genome analyses [12,13,37]. It is likely that some of the observed variations among genes encoding GTs in *H. somni *and other *Pasteurellaceae *members is due to recombination events and/or selective pressure. Furthermore, variation in the composition and structure of the LOSs of strains 2336 and 129Pt may, in part, be due to different GT genes they have acquired or lost. In view of this, the role of new GT genes putatively involved in LOS biosynthesis and phase variation that have been identified in this study needs to be investigated.

Several types of two-partner secretion (TPS) pathways have been identified and characterized in Gram-negative bacteria [48]. Filamentous hemagglutinins (Fha), consisting of a membrane-anchored protein (FhaC), which is involved in the activation/secretion of the cognate hemagglutinin/adhesin (FhaB), are prototypes of two-partner virulence systems. Homologs of FhaB and FhaC that possibly play a role in pathogenesis have been found in several members of the genera *Bordetella, Haemophilus, Proteus*, and *Pasteurella*, [49-53]. Among the 4 loci containing *fha *homologs in strain 2336, locus II appeared to be an acquisition mediated by a transposon and locus I appeared to be an acquisition due to homologous recombination. It appears that strain 129Pt has lost an *fhaB *homolog due to bacteriophage excision (Locus IV). It is possible that *fhaB *homologs in locus I of strain 2336 are paralogs, as are the *fhaB *homologs in locus IV. Together, these genes represent a large collection of *fhaB *and *fhaC *homologs in a single genome. The presence of multiple *fhaB *homologs in strain 2336 may, in part, be responsible for the serum resistance of this strain, in contrast to strain 129Pt, which does not contain full length *fhaB *homologs and is serum-sensitive [10]. Structural and functional studies of N-terminal fragments of FHA from *B. pertussis *and HpmA from *P*. *mirabilis *have indicated that the proteins form a right-handed parallel β-helix [52-54]. Several residues that mediate the interaction of *B*. *pertusis *FHA with its cognate FhaC, and facilitate secretion, have also been identified [54]. Although *H. somni *FhaB homologs have some features in common with FHA of *B. pertussis *and HpmA of *P*. *mirabilis*, a distinct region is the direct repeat 2 Fic domain in the FhaB homolog in locus III of strain 2336 that has been shown to induce cytotoxicity in human HeLa cells, bovine turbinate cells, and bovine alveolar type 2 cells [55,56]. However, the secretion determinants of these proteins and the role of FhaC proteins in their secretion remain unknown.

Subtilases ([GenBank:COG1404]; subtilisin-like serine proteases) are a large superfamily of functionally diverse endo- and exo-peptidases that occur in prokaryotes and eukaryotes [57]. Bacterial subtilisins may have a role in pathogenesis besides facilitating protein degradation and nutrient acquisition [58]. However, subtilisin-like serine proteases from members of the *Pasteurellaceae *have not been characterized previously. The presence of a gene encoding a putative subtilase whose homologs were not found in other members of the *Pasteurellaceae *was yet another example of HGT in strain 2336. Although *H. ducreyi *strain 35000HP contains genes ([GenBank:HD1094] and [GenBank:HD1278]) encoding serine proteases that belong to the D-H-S family, they are unrelated to each other and to [GenBank:HSM_188]. In *Agrobacterium tumefaciens*, genes encoding AAA-ATPase and subtilisin-like serine protease have been shown to be functionally related and this pair has been proposed to constitute a toxin-antitoxin system that contributes to stability of plasmid pTiC58 [59]. A conjugative megaplasmid-encoded subtilase has been shown to be a virulence factor in *E. coli *and it has been suggested this toxin activates a V-ATPase in Vero cells [60,61]. Whether the ATPase-subtilisin pair identified in this study is transcriptionally and functionally coupled, and whether the protease gene contributes to the pathogenicity of strain 2336, has yet to be determined.

The ability to acquire and metabolize iron is an important determinant of bacterial survival and adaptability. In some bacteria, genes that facilitate iron uptake have been shown to be acquired by horizontal transfer [62]. Several members of the *Pasteurellaceae *possess special outer membrane protein (OMP) receptors consisting of two unrelated transferrin-binding proteins, TbpA and TbpB, which facilitate acquisition of transferrin-bound iron from their hosts [63]. *H. somni *strain 649 has a TbpA-TbpB receptor system that acquires iron only from bovine transferrin, and a second, probably redundant, TbpA2 receptor that can acquire iron from bovine, caprine, or ovine transferrins [64]. From genomic analyses, it is apparent that *H. somni *strains possess multiple genes for the acquisition of iron. Horizontal transfer and clustering of genes related to iron metabolism is indicative of enrichment and adaptation of pathogenic *H. somni *to different niches within its natural host. Furthermore, products of one or more of these genes may facilitate binding to transferrins/lactoferrins of different host species and such a gene repertoire could enhance the ability of this bacterium to survive in a variety of ruminants. Studies using a mouse model have suggested the role of bovine transferrin and lactoferrin in increasing the virulence of strain 2336 [65].

Many bacterial pathogens contain surface proteins that facilitate adhesion to and/or invasion of the host mucosal barriers [66,67]. Some of these proteins may also be involved in bacterial aggregation to form biofilms and their evasion of the host's innate immune system [67,68]. At least three major categories of bacterial adherence proteins have been identified, two of which are hair-like structures called pili and non-pilus-associated proteins called adhesins. The plasmid-encoded YadA is a prototype non-pilus-associated protein that has been well characterized [69]. Mutation or deletion of the *yadA *homolog can reduce virulence in pathogenic bacteria [70,71]. Large adhesins in some bacteria are associated with ABC transporters and may be involved in biofilm formation [72]. A transposon mutagenesis approach has implicated several genes, including those encoding filamentous haemagglutinins, in *H. somni *biofilm formation [73]. *H. somni *also contains genes putatively involved in adhesin synthesis/transport, pilus formation, and quorum sensing (*e.g*., *luxS*), but their role in facilitating biofilm formation remains to be investigated.

Veterinarians use several antibiotics to treat *H. somni *infections and feedlot cattle enterprises frequently rely on tetracyclines for prophylaxis as well as growth promotion [1,74,75]. Although not common, *H. somni *resistance to tetracycline has been reported [76,77]. Copper and zinc are often included in commercial cattle diets to achieve optimal growth and reproduction. Emergence of copper/zinc resistance in bacteria of animal origin has been documented and attributed to the excessive presence of these metals in livestock feed [78]. Furthermore, the occurrence of genes related to metal and antibiotic resistance on integrative/conjugative elements and their horizontal co-transfer has been noted previously [79,80]. In view of these observations, it was not surprising to find a GI containing genes putatively involved in copper, zinc, and tetracycline resistance in strain 2336.

Transcriptional regulators play crucial roles in bacterial functions and they have been classified into a number of families [81]. The homologs of [GenBank:HSM_0806] (LysR, [NCBI:CLSK797597] cluster) in *H. influenzae *and *P. dagmatis *are associated with genes encoding proteins involved in fatty acid metabolism (*e.g*., acetyl-CoA acetyltransferase, 3-oxoacid CoA- transferase, and fatty acid transporters). Therefore, this cluster may represent a novel class of metabolic regulators within the LysR family. Most members of the [NCBI:PRK13756] cluster are involved in regulation of antibiotic resistance genes [81]. Homologs of [GenBank:HSM_1734] and [GenBank:HSM_1735] among members of the *Pasteurellaceae *encode tetracycline resistance and are associated with mobile genetic elements [82-86]. Homologs of [GenBank:HSM_1736] and [GenBank:HSM_1737] in other bacteria are known to be horizontally transferred and may mediate resistance to antibiotics [87]. Furthermore, homologs of [GenBank:HSM_1192] and [GenBank:HSM_1193] are predicted to be involved in multidrug resistance [88,89]. In summary, it appears that strain 2336 contained at least three different systems related to antibiotic resistance. Although the functional role of these genes remains to be established, their similarity to metal/antibiotic resistance genes associated with mobile genetic elements in other members of the *Pasteurellaceae *is clinically significant.

From genome comparisons, it appears that there is no correlation between chromosome size and the number of tRNA genes (the genomes of *H. somni *2336, *H. somni *129Pt, *H. influenzae *86-028NP, *H. ducreyi *35000HP, and *P. multocida *Pm70 contain 49, 49, 58, 46, and 57 tRNA genes, respectively). Whether the lower number of tRNA genes found in *H. somni *strains is due to disruptive integration of bacteriophages into tRNA genes (as in 'bacteriophage disruption of tRNA genes in *Lactobacillus johnsonii*' [90]) or is a result of compensatory gene loss in lieu of acquisition of new genes (as in 'genome reduction in pathogenic and symbiotic bacteria' [91]) is unknown. Nevertheless, comparison of the chromosomes of strains 129Pt and 2336 bolsters the proposition that prophages and transposons have played a major role in creating genomic diversity and phenotypic variability in the two strains. It is also apparent that strains 2336 and 129Pt have independently and intermittently acquired and lost genes since their divergence from a common ancestor, and that the net gain in strain 129Pt is less than the net gain in strain 2336.

## Conclusions

*H. somni *strain 2336 contains a larger chromosome when compared to other *Haemophilus *and *Histophilus *strains whose genome sequences are available. Several regions that resemble the pathogenicity islands of other virulent bacteria are present in strain 2336. There is evidence to suggest that most of these regions were acquired by HGT mechanisms, whereas similar regions were not found in the commensal strain 129Pt. Although previous studies have discovered the genetic basis for some of the phenotypic dissimilarities between strains 2336 and 129Pt, complete genome sequence analyses have provided a comprehensive account of innate and acquired genetic traits. Furthermore, comparisons of the genomes of strains 2336 and 129Pt have contributed to our understanding of the biology and pathogenic evolution of these bacteria. The post-genomic era for *H. somni *poses new challenges and opportunities in terms of functional characterization of genes and deciphering their roles in colonization, survival, and pathogenesis. Continued analyses of the genomes of *H. somni *strains and comparison with newly sequenced genomes of other bacteria should enhance the current knowledge on virulence mechanisms. Nevertheless, the results from this study are expected to facilitate the development of improved diagnostic tests for and vaccines against *H. somni*.

## Authors' contributions

SS performed the annotation using RAST, planned the comparative analysis, generated all the figures, and drafted most of the manuscript. JFC, AJD, and AFG contributed to whole genome comparisons and identification of putative virulence genes. MC, JG, JO, JZ, and GB contributed to the whole genome shotgun sequencing of strain 2336 at OUHSC. DB, OC, JCD, CSH, and RT contributed to the finishing of the genome of strain 2336 at LANL and participated in the study design and comparative analysis of the two genomes. TJI and DWD conceived the study, participated in genome analyses, and helped draft the manuscript. All authors read and approved the final manuscript.

## Supplementary Material

Additional file 1 **Additional file 1 List of *H. somni *strain 2336 specific genes**. This table lists the strain-specific genes found in *H. somni *strain 2336. This data was obtained by cross-comparison of the genomes of strains 2336 and 129Pt using blastn. Click here for file

Additional file 2 **Additional file 2 List of *H. somni *strain 129Pt specific genes**. This table lists the strain-specific genes found in *H. somni *strain 129Pt. This data was obtained by cross-comparison of the genomes of strains 129Pt and 2336 using blastn. Click here for file
