# Supplementary material for: Horizontal gene transfer in Histophilus somni and its role in the evolution of pathogenic strain 2336, as determined by comparative genomic analyses
Source: BMC Genomics. 2011 Nov 23;12:570. doi: 10.1186/1471-2164-12-570 (PMC3339403; doi:10.1186/1471-2164-12-570)
Supplement: Additional file 1 — List of H. somni strain 2336 specific genes. This table lists the strain-specific genes found in H. somni strain 2336. This data was obtained by cross-comparison of the genomes of strains 2336 and 129Pt using blastn. [file 1471-2164-12-570-S1.DOCX]

**Additional file 1. List of *H. somni* strain 2336 specific genes**

**Product Name**  **Start**  **End**  **Length**  **Locus_tag**

hypothetical protein [GenBank:HSM_0225] 243787 244248 153 [GenBank:HSM_0225]

phage head completion 244245 244697 150 [GenBank:HSM_0226]

phage small terminase subunit 244690 245571 293 [GenBank:HSM_0227]

P2 family phage major capsid protein 245600 246631 343 [GenBank:HSM_0228]

hypothetical protein [GenBank:HSM_0229] 246641 247066 141 [GenBank:HSM_0229]

phage capsid scaffolding 247017 247538 173 [GenBank:HSM_0230]

hypothetical protein [GenBank:HSM_0231] 247701 249488 595 [GenBank:HSM_0231]

PBSX family phage portal protein 249499 250527 342 [GenBank:HSM_0232]

phage transcriptional activator Ogr/delta 250583 250861 92 [GenBank:HSM_0233]

hypothetical protein [GenBank:HSM_0234] 250962 251357 131 [GenBank:HSM_0234]

N-6 DNA methylase 251354 252739 461 [GenBank:HSM_0235]

hypothetical protein [GenBank:HSM_0236] 252756 253286 176 [GenBank:HSM_0236]

hypothetical protein [GenBank:HSM_0237] 253301 255490 729 [GenBank:HSM_0237]

hypothetical protein [GenBank: HSM_0238] 255487 255756 89 [GenBank:HSM_0238]

hypothetical protein [GenBank:HSM_0239] 255749 256237 162 [GenBank:HSM_0239]

hypothetical protein [GenBank:HSM_0240] 256389 256667 92 [GenBank:HSM_0240]

DNA methylase N-4/N-6 256736 257500 254 [GenBank:HSM_0241]

domain-containing protein

hypothetical protein [GenBank:HSM_0242] 257511 257972 153 [GenBank:HSM_0242]

hypothetical protein [GenBank:HSM_0243] 258443 258724 93 [GenBank:HSM_0243]

hypothetical protein [GenBank:HSM_0244] 258693 258962 89 [GenBank:HSM_0244]

hypothetical protein [GenBank:HSM_0245] 259257 259694 145 [GenBank:HSM_0245]

hypothetical protein [GenBank:HSM_0246] 261096 261314 72 [GenBank:HSM_0246]

hypothetical protein [GenBank:HSM_0247] 262158 262610 150 [GenBank:HSM_0247]

DNA-binding protein 262646 263011 121 [GenBank:HSM_0248]

addiction module killer protein 263373 263696 107 [GenBank:HSM_0249]

putative transcriptional regulator 263689 263988 99 [GenBank:HSM_0250]

hemolysin activation/secretion 279733 281478 581 [GenBank:HSM_0267]

protein-like protein (FhaC)

filamentous haemagglutinin 281512 288630 2372 [GenBank:HSM_0268]

outer membrane protein (FhaB)

hypothetical protein [GenBank:HSM_0269] 288599 288940 113 [GenBank:HSM_0269]

filamentous haemagglutinin 289047 292526 1159 [GenBank:HSM_0270]

outer membrane protein (FhaB)

hypothetical protein [GenBank:HSM_0271] 292546 293073 175 [GenBank:HSM_0271]

filamentous haemagglutinin 293222 294907 561 [GenBank:HSM_0272]

outer membrane protein (FhaB)

hypothetical protein [GenBank:HSM_0273] 294885 295271 128 [GenBank:HSM_0273]

filamentous haemagglutinin 295335 296903 522 [GenBank:HSM_0274]

outer membrane protein (FhaB)

hypothetical protein [GenBank:HSM_0275] 296905 297255 116 [GenBank:HSM_0275]

hypothetical protein [GenBank:HSM_0276] 297372 297707 111 [GenBank:HSM_0276]

hypothetical protein [GenBank:HSM_0295] 318940 319335 131 [GenBank:HSM_0295]

hypothetical protein [GenBank:HSM_0445] 519037 519393 118 [GenBank:HSM_0445]

hypothetical protein [GenBank:HSM_0447] 522375 522641 88 [GenBank:HSM_0447]

hypothetical protein [GenBank:HSM_0487] 564631 564834 67 [GenBank:HSM_0487]

hypothetical protein [GenBank:HSM_0488] 564878 565087 69 [GenBank:HSM_0488]

putative transcriptional regulator 565134 566492 452 [GenBank:HSM_0489]

hypothetical protein [GenBank:HSM_0528] 606234 606383 49 [GenBank:HSM_0528]

hypothetical protein [GenBank:HSM_0530] 607181 607300 39 [GenBank:HSM_0530]

transposase 607402 607560 52 [GenBank:HSM_0531]

transposase 607593 607736 47 [GenBank:HSM_0532]

hypothetical protein [GenBank:HSM_0593] 692750 693163 137 [GenBank:HSM_0593]

putative transposase 693192 694253 353 [GenBank:HSM_0594]

hypothetical protein [GenBank:HSM_0595] 694739 695035 98 [GenBank:HSM_0595]

DNA-cytosine methyltransferase 695021 696139 372 [GenBank:HSM_0596]

Type II site-specific deoxyribonuclease 696139 697188 349 [GenBank:HSM_0597]

putative stress-sensitive restriction system protein 697189 699078 629 [GenBank:HSM_0598]

XRE family transcriptional regulator 699183 699461 92 [GenBank:HSM_0599]

hypothetical protein [GenBank:HSM_0602] 700602 701042 146 [GenBank:HSM_0602]

hypothetical protein [GenBank:HSM_0603] 701055 701204 49 [GenBank:HSM_0603]

hypothetical protein [GenBank:HSM_0609] 705119 706027 302 [GenBank:HSM_0609]

N-6 DNA methylase 706031 709363 1110 [GenBank:HSM_0610]

restriction modification enzyme 709456 709941 161 [GenBank:HSM_0611]

hypothetical protein [GenBank:HSM_0643] 745806 746348 180 [GenBank:HSM_0643]

ABC transporter related 746757 747233 158 [GenBank:HSM_0644]

putative phage repressor 747563 747808 81 [GenBank:HSM_0645]

hypothetical protein [GenBank:HSM_0646] 748000 748527 175 [GenBank:HSM_0646]

transposase 748861 750849 662 [GenBank:HSM_0647]

helix-turn-helix domain-containing protein 750853 751728 291 [GenBank:HSM_0648]

hypothetical protein [GenBank:HSM_0649] 751951 752166 71 [GenBank:HSM_0649]

hypothetical protein [GenBank:HSM_0650] 752175 752369 64 [GenBank:HSM_0650]

hypothetical protein [GenBank:HSM_0651] 752606 753223 205 [GenBank:HSM_0651]

hypothetical protein [GenBank:HSM_0652 753390 753710 106 [GenBank:HSM_0652]

hypothetical protein [GenBank:HSM_0653] 754070 754237 55 [GenBank:HSM_0653]

hypothetical protein [GenBank:HSM_0654] 754404 754796 130 [GenBank:HSM_0654]

hypothetical protein [GenBank:HSM_0655] 754904 755254 116 [GenBank:HSM_0655]

hypothetical protein [GenBank:HSM_0656 755381 755755 124 [GenBank:HSM_0656]

hypothetical protein [GenBank:HSM_0657] 755742 756152 136 [GenBank:HSM_0657]

hypothetical protein [GenBank:HSM_0658] 756212 756706 164 [GenBank:HSM_0658]

hypothetical protein [GenBank:HSM_0659] 756891 757319 142 [GenBank:HSM_0659]

N-acetylmuramoyl-L-alanine amidase 757402 757938 178 [GenBank:HSM_0660]

hypothetical protein [GenBank:HSM_0661] 758081 758323 80 [GenBank:HSM_0661]

hypothetical protein [GenBank:HSM_0662] 758320 758574 84 [GenBank:HSM_0662]

hypothetical protein [GenBank:HSM_0663] 758685 758939 84 [GenBank:HSM_0663]

hypothetical protein [GenBank:HSM_0664] 758939 759193 84 [GenBank:HSM_0664]

hypothetical protein [GenBank:HSM_0665] 759194 759697 167 [GenBank:HSM_0665]

phage uncharacterized protein 759800 761443 547 [GenBank:HSM_0666]

hypothetical protein [GenBank:HSM_0667] 761447 763105 552 [GenBank:HSM_0667]

SPP1 family phage head morphogenesis protein 763092 764366 424 [GenBank:HSM_0668]

phage virion morphogenesis protein 764940 765425 161 [GenBank:HSM_0669]

putative I protein 765686 766726 346 [GenBank:HSM_0670]

bacteriophage mu T-like protein 766731 767657 308 [GenBank:HSM_0671]

hypothetical protein [GenBank:HSM_0672] 767701 768171 156 [GenBank:HSM_0672]

hypothetical protein [GenBank:HSM_0673] 768174 768608 144 [GenBank:HSM_0673]

hypothetical protein [GenBank:HSM_0674] 768520 769233 237 [GenBank:HSM_0674]

hypothetical protein [GenBank:HSM_0675] 769230 769412 60 [GenBank:HSM_0675]

bacteriophage Mu tail sheath family protein 769405 770820 471 [GenBank:HSM_0676]

hypothetical protein [GenBank:HSM_0677] 770830 771204 124 [GenBank:HSM_0677]

hypothetical protein [GenBank:HSM_0678] 771204 771575 123 [GenBank:HSM_0678]

hypothetical protein [GenBank:HSM_0679] 771838 774255 805 [GenBank:HSM_0679]

putative phage virion protein 774258 775589 443 [GenBank:HSM_0680]

bacteriophage Mu P family protein 775579 776724 381 [GenBank:HSM_0681]

phage baseplate assembly protein V 776724 777380 218 [GenBank:HSM_0682]

phage GP46 family protein 777459 777809 116 [GenBank:HSM_0683]

baseplate J family protein 777823 778887 354 [GenBank:HSM_0684]

hypothetical protein [GenBank:HSM_0685] 778887 779474 195 [GenBank:HSM_0685]

phage tail collar domain-containing protein 779478 781436 652 [GenBank:HSM_0686]

hypothetical protein [GenBank:HSM_0687] 781433 782047 204 [GenBank:HSM_0687]

hypothetical protein [GenBank:HSM_0688 782040 782534 164 [GenBank:HSM_0688]

Putative C5 methylase (MAV1virus-like) 782902 783660 252 [GenBank:HSM_0689]

hypothetical protein [GenBank:HSM_0690 783647 784483 278 [GenBank:HSM_0690]

hypothetical protein [GenBank:HSM_0691] 784504 784692 62 [GenBank:HSM_0691]

hypothetical protein [GenBank:HSM_0692] 784696 785010 104 [GenBank:HSM_0692]

ABC transporter related 785160 786344 394 [GenBank:HSM_0693]

binding-protein-dependent transport systems 786356 787276 306 [GenBank:HSM_0694]

inner membrane component

alkaline phosphatase 787286 788209 307 [GenBank:HSM_0695]

extracellular solute-binding protein 788322 789959 545 [GenBank:HSM_0696]

extracellular solute-binding protein 790118 791773 551 [GenBank:HSM_0697]

DNA-cytosine methyltransferase 917815 918786 323 [GenBank:HSM_0801]

hypothetical protein [GenBank:HSM_0802] 918797 919540 247 [GenBank:HSM_0802]

putative HTH-type transcriptional regulator 924369 925268 299 [GenBank:HSM_0806]

3-oxoacid CoA-transferase, A subunit 925565 926212 215 [GenBank:HSM_0807]

3-oxoacid CoA-transferase, B subunit 926230 926889 219 [GenBank:HSM_0808]

short-chain fatty acids transporter 926900 928243 447 [GenBank:HSM_0809]

acetyl-CoA acetyltransferase 928335 929516 393 [GenBank:HSM_0810]

3-hydroxybutyrate dehydrogenase 929544 930302 252 [GenBank:HSM_0811]

citrate transporter 930373 931758 461 [GenBank:HSM_0812]

two component transcriptional regulator 936328 937056 242 [GenBank:HSM_0817]

ribokinase-like domain-containing protein 937089 938084 331 [GenBank:HSM_0818]

ketose-bisphosphate aldolase 938071 938955 294 [GenBank:HSM_0819]

hypothetical protein [GenBank:HSM_0820] 938946 939626 226 [GenBank:HSM_0820]

periplasmic binding protein/LacI 939733 940662 309 [GenBank:HSM_0821]

transcriptional regulator

monosaccharide-transporting ATPase 940717 941685 322 [GenBank:HSM_0822]

ABC transporter related 941708 943216 502 [GenBank:HSM_0823]

integral membrane sensor hybrid histidine kinase 943549 945978 809 [GenBank:HSM_0824]

hypothetical protein [GenBank:HSM_0848] 982522 982908 128 [GenBank:HSM_0848]

hypothetical protein [GenBank:HSM_0856] 995904 996848 314 [GenBank:HSM_0856]

phage integrase family protein 1001039 1002094 351 [GenBank:HSM_0860]

hypothetical protein [GenBank:HSM_0862] 1003733 1004233 166 [GenBank:HSM_0862]

hypothetical protein [GenBank:HSM_0863] 1004294 1004722 142 [GenBank:HSM_0863]

hypothetical protein [GenBank:HSM_0864] 1005041 1005679 212 [GenBank:HSM_0864]

hypothetical protein [GenBank:HSM_0865] 1005832 1006158 108 [GenBank:HSM_0865]

hypothetical protein [GenBank:HSM_0866] 1006155 1006592 145 [GenBank:HSM_0866]

hypothetical protein [GenBank:HSM_0867] 1006589 1006873 94 [GenBank:HSM_0867]

putative antirepressor protein 1006960 1007799 279 [GenBank:HSM_0868]

hypothetical protein [GenBank:HSM_0869] 1008591 1009388 265 [GenBank:HSM_0869]

hypothetical protein [GenBank:HSM_0870] 1009789 1009974 61 [GenBank:HSM_0870]

hypothetical protein [GenBank:HSM_0871] 1010153 1010518 121 [GenBank:HSM_0871]

hypothetical protein [GenBank:HSM_0872] 1010620 1011249 209 [GenBank:HSM_0872]

hypothetical protein [GenBank:HSM_0873] 1011246 1011908 220 [GenBank:HSM_0873]

KilA domain-containing protein 1011972 1012604 210 [GenBank:HSM_0874]

hypothetical protein [GenBank:HSM_0875] 1012882 1013709 275 [GenBank:HSM_0875]

hypothetical protein [GenBank:HSM_0876] 1014768 1014989 73 [GenBank:HSM_0876]

hypothetical protein [GenBank:HSM_0877] 1015001 1015189 62 [GenBank:HSM_0877]

addiction module killer protein 1015606 1015902 98 [GenBank:HSM_0878]

putative transcriptional regulator 1015904 1016200 98 [GenBank:HSM_0879]

hypothetical protein [GenBank:HSM_0880] 1016228 1017232 334 [GenBank:HSM_0880]

putative prophage repressor 1017329 1018015 228 [GenBank:HSM_0881]

regulatory protein 1018118 1018345 75 [GenBank:HSM_0882]

hypothetical protein [GenBank:HSM_0892] 1024492 1024896 134 [GenBank:HSM_0892]

putative antirepressor protein 1032458 1033333 291 [GenBank:HSM_0901]

hypothetical protein [GenBank:HSM_0914] 1048140 1048766 208 [GenBank:HSM_0914]

phage virion morphogenesis 1053004 1053252 82 [GenBank:HSM_0921]

(putative tail completion) protein

hypothetical protein [GenBank:HSM_0922] 1053507 1053884 125 [GenBank:HSM_0922]

hypothetical protein [GenBank:HSM_0923] 1053982 1054251 89 [GenBank:HSM_0923]

hypothetical protein [GenBank:HSM_1039] 1197197 1197430 77 [GenBank:HSM_1039]

hypothetical protein [GenBank:HSM_1056] 1215662 1216675 337 [GenBank:HSM_1056]

hemolysin activation/secretion 1248725 1250077 450 [GenBank:HSM_1089]

protein-like protein (FhaC)

filamentous haemagglutinin 1250109 1255376 1755 [GenBank:HSM_1090]

outer membrane protein (FhaB)

bacteriophage replication gene A 1302900 1305263 787 [GenBank:HSM_1131]

hypothetical protein [GenBank:HSM_1132] 1305282 1305575 97 [GenBank:HSM_1132]

hypothetical protein [GenBank:HSM_1133] 1305572 1305925 117 [GenBank:HSM_1133]

hypothetical protein [GenBank:HSM_1134] 1305941 1306231 96 [GenBank:HSM_1134]

hypothetical protein [GenBank:HSM_1135] 1306630 1306875 81 [GenBank:HSM_1135]

putative phage repressor 1307015 1307704 229 [GenBank:HSM_1136]

phage late control D family protein 1307987 1309162 391 [GenBank:HSM_1137]

phage P2 GpU family protein 1309159 1309602 147 [GenBank:HSM_1138]

phage tail E family protein 1309807 1310115 102 [GenBank:HSM_1139]

phage major tail tube protein 1310143 1310652 169 [GenBank:HSM_1140]

phage tail sheath protein 1310663 1311916 417 [GenBank:HSM_1141]

hypothetical protein [GenBank:HSM_1142] 1312129 1312377 82 [GenBank:HSM_1142]

addiction module antitoxin 1312377 1312640 87 [GenBank:HSM_1143]

hypothetical protein [GenBank:HSM_1144] 1312855 1313043 62 [GenBank:HSM_1144]

hypothetical protein [GenBank:HSM_1145] 1313046 1313522 158 [GenBank:HSM_1145]

hypothetical protein [GenBank:HSM_1146] 1313515 1314141 208 [GenBank:HSM_1146]

hypothetical protein [GenBank:HSM_1147] 1314204 1314455 83 [GenBank:HSM_1147]

hypothetical protein [GenBank:HSM_1148] 1314439 1314756 105 [GenBank:HSM_1148]

phage tail collar domain-containing protein 1314792 1316891 699 [GenBank:HSM_1149]

phage tail protein I 1316937 1317491 184 [GenBank:HSM_1150]

baseplate J family protein 1317481 1318395 304 [GenBank:HSM_1151]

GPW/gp25 family protein 1318379 1318735 118 [GenBank:HSM_1152]

phage baseplate assembly protein V 1318735 1319361 208 [GenBank:HSM_1153]

hypothetical protein [GenBank:HSM_1154] 1319575 1319922 115 [GenBank:HSM_1154]

hypothetical protein [GenBank:HSM_1155] 1319910 1320194 94 [GenBank:HSM_1155]

TP901 family phage tail tape measure protein 1320349 1322847 832 [GenBank:HSM_1156]

phage virion morphogenesis protein 1323613 1324074 153 [GenBank:HSM_1157]

P2 phage tail completion R family protein 1324087 1324527 146 [GenBank:HSM_1158]

hypothetical protein [GenBank:HSM_1159] 1324885 1325343 152 [GenBank:HSM_1159]

hypothetical protein [GenBank:HSM_1161] 1325856 1326080 74 [GenBank:HSM_1161]

phage tail X family protein 1326081 1326296 71 [GenBank:HSM_1162]

phage head completion 1326296 1326808 170 [GenBank:HSM_1163]

phage small terminase subunit 1326912 1327565 217 [GenBank:HSM_1164]

P2 family phage major capsid protein 1327577 1328629 350 [GenBank:HSM_1165]

phage capsid scaffolding 1328653 1329477 274 [GenBank:HSM_1166]

putative terminase ATPase subunit 1329641 1329985 114 [GenBank:HSM_1167]

TonB-dependent hemoglobin/transferrin/ 1330234 1333344 1036 [GenBank:HSM_1168]

lactoferrin family receptor

23S rRNA (uracil-5-)-methyltransferase RumA 1340138 1340428 96 [GenBank:HSM_1177]

hypothetical protein [GenBank:HSM_1178] 1340805 1341248 147 [GenBank:HSM_1178]

hypothetical protein [GenBank: HSM_1181] 1342054 1342632 192 [GenBank:HSM_1181]

nucleic acid-binding protein 1342706 1343545 279 [GenBank:HSM_1182]

hypothetical protein [GenBank:HSM_1183] 1343736 1344005 89 [GenBank:HSM_1183]

hypothetical protein [GenBank:HSM_1184] 1343931 1344473 180 [GenBank:HSM_1184]

hypothetical protein [GenBank:HSM_1185] 1344682 1344798 38 [GenBank:HSM_1185]

hypothetical protein [GenBank:HSM_1186] 1344940 1345881 313 [GenBank:HSM_1186]

hypothetical protein [GenBank:HSM_1187] 1346067 1346642 191 [GenBank:HSM_1187]

hypothetical protein [GenBank:HSM_1188] 1346768 1347130 120 [GenBank:HSM_1188]

hypothetical protein [GenBank:HSM_1189] 1347140 1348855 571 [GenBank:HSM_1189]

relaxase/mobilization nuclease family protein 1349004 1349972 322 [GenBank:HSM_1190]

TetR family transcriptional regulator 1350057 1350629 190 [GenBank:HSM_1191]

ABC transporter, ATP-binding/permease protein 1350716 1351171 151 [GenBank:HSM_1192]

ABC-type cobalt transport system 1351225 1351563 112 [GenBank:HSM_1193]

ATPase component-like protein

hypothetical protein [GenBank:HSM_1351] 1534340 1534759 139 [GenBank:HSM_1351]

hypothetical protein [GenBank:HSM_1443] 1650043 1650309 88 [GenBank:HSM_1443]

filamentation induced by cAMP protein Fic 1650254 1650916 220 [GenBank:HSM_1444]

virulence-associated protein D (VapD) region 1653426 1653707 93 [GenBank:HSM_1448]

hypothetical protein [GenBank:HSM_1483 1688789 1688941 50 [GenBank:HSM_1483]

filamentous haemagglutinin 1701024 1713311 4095 [GenBank:HSM_1489]

outer membrane protein (FhaB)

hemolysin activation/secretion 1713336 1715096 586 [GenBank:HSM_1490]

protein-like protein (FhaC)

putative transposase 1747439 1748563 374 [GenBank:HSM_1521]

hypothetical protein [GenBank:HSM_1522] 1750248 1750766 172 [GenBank:HSM_1522]

hypothetical protein [GenBank:HSM_1523] 1750792 1751304 170 [GenBank:HSM_1523]

hypothetical protein [GenBank:HSM_1524] 1751470 1752072 200 [GenBank:HSM_1524]

hypothetical protein [GenBank:HSM_1525] 1752082 1752618 178 [GenBank:HSM_1525]

hypothetical protein [GenBank:HSM_1526] 1752864 1753844 326 [GenBank:HSM_1526]

hypothetical protein [GenBank:HSM_1527] 1754084 1754551 155 [GenBank:HSM_1527]

hypothetical protein [GenBank: HSM_1570] 1806382 1806894 170 [GenBank:HSM_1570]

Abi family protein 1864717 1865697 326 [GenBank:HSM_1616]

filamentation induced by cAMP protein Fic 1867032 1867592 186 [GenBank:HSM_1618]

type I restriction-modification system, M subunit 1867646 1869259 537 [GenBank:HSM_1619]

hypothetical protein [GenBank:HSM_1630] 1875907 1876092 61 [GenBank:HSM_1630]

hypothetical protein [GenBank:HSM_1633] 1877974 1878402 142 [GenBank:HSM_1633]

hypothetical protein [GenBank:HSM_1634] 1879063 1879773 236 [GenBank:HSM_1634]

hypothetical protein [GenBank:HSM_1635] 1879770 1880156 128 [GenBank:HSM_1635]

hypothetical protein [GenBank:HSM_1636] 1880159 1880308 49 [GenBank:HSM_1636]

hypothetical protein [GenBank:HSM_1637] 1880376 1880621 81 [GenBank:HSM_1637]

filamentous haemagglutinin 1880626 1882686 686 [GenBank:HSM_1638]

outer membrane protein (FhaB)

hypothetical protein [GenBank:HSM_1639] 1883603 1884052 149 [GenBank:HSM_1639]

hypothetical protein [GenBank:HSM_1640] 1884363 1884803 146 [GenBank:HSM_1640]

filamentous haemagglutinin 1884812 1886914 700 [GenBank:HSM_1641]

outer membrane protein (FhaB)

hypothetical protein [GenBank:HSM_1642] 1887064 1887519 151 [GenBank:HSM_1642]

filamentous haemagglutinin 1887521 1887772 83 [GenBank:HSM_1643]

outer membrane protein (FhaB)

hypothetical protein [GenBank:HSM_1644] 1888067 1888423 118 [GenBank:HSM_1644]

hypothetical protein [GenBank:HSM_1645] 1888675 1888926 83 [GenBank:HSM_1645]

filamentous haemagglutinin 1888923 1890218 431 [GenBank:HSM_1646]

outer membrane protein (FhaB)

filamentous haemagglutinin 1890444 1892567 707 [GenBank:HSM_1647]

outer membrane protein (FhaB)

hypothetical protein [GenBank:HSM_1648] 1892583 1892975 130 [GenBank:HSM_1648]

hypothetical protein [GenBank:HSM_1649] 1892975 1893256 93 [GenBank:HSM_1649]

hypothetical protein [GenBank:HSM_1650] 1893295 1893780 161 [GenBank:HSM_1650]

filamentous haemagglutinin 1893785 1902019 2744 [GenBank:HSM_1651]

outer membrane protein (FhaB)

hemolysin activation/secretion 1901988 1902182 64 [GenBank:HSM_1652]

protein-like protein (FhaC)

hypothetical protein [GenBank:HSM_1653] 1902170 1902466 98 [GenBank:HSM_1653]

type III restriction enzyme, res subunit 1913858 1914580 240 [GenBank:HSM_1665]

type III restriction protein res subunit 1914598 1918695 1365 [GenBank:HSM_1666]

hypothetical protein [GenBank:HSM_1675] 1924840 1925814 324 [GenBank:HSM_1675]

hypothetical protein [GenBank:HSM_1677] 1928343 1928921 192 [GenBank:HSM_1677]

hypothetical protein [GenBank:HSM_1678] 1929653 1930090 145 [GenBank:HSM_1678]

integrase catalytic region 1931538 1932512 324 [GenBank:HSM_1680]

putative type I restriction enzyme M subunit 1932585 1933382 265 [GenBank:HSM_1681]

hypothetical protein [GenBank:HSM_1682] 1933455 1934201 248 [GenBank:HSM_1682]

hypothetical protein [GenBank:HSM_1685] 1936083 1936925 280 [GenBank:HSM_1685]

hypothetical protein [GenBank:HSM_1686] 1937003 1937398 131 [GenBank:HSM_1686]

hypothetical protein [GenBank:HSM_1688] 1937814 1938167 117 [GenBank:HSM_1688]

unknown phage protein 1942617 1943087 156 [GenBank:HSM_1693]

hypothetical protein [GenBank:HSM_1694] 1943108 1943449 113 [GenBank:HSM_1694]

hypothetical protein [GenBank:HSM_1715] 1959973 1960233 86 [GenBank:HSM_1715]

hypothetical protein [GenBank:HSM_1718] 1961221 1961901 226 [GenBank:HSM_1718]

aldo/keto reductase 1967711 1968478 255 [GenBank:HSM_1723]

hypothetical protein [GenBank:HSM_1724] 1968477 1968797 106 [GenBank:HSM_1724]

hypothetical protein [GenBank:HSM_1725] 1968979 1969434 151 [GenBank:HSM_1725]

multicopper oxidase type 3 1969449 1970996 515 [GenBank:HSM_1726]

alcohol dehydrogenase 1971086 1971241 51 [GenBank:HSM_1727]

MerR family transcriptional regulator 1971664 1972068 134 [GenBank:HSM_1728]

hypothetical protein [GenBank:HSM_1729] 1972283 1972600 105 [GenBank:HSM_1729]

multicopper oxidase type 3 1972853 1974457 534 [GenBank:HSM_1730]

heavy metal translocating P-type ATPase 1974826 1977018 730 [GenBank:HSM_1731]

hypothetical protein [GenBank:HSM_1732] 1977326 1978072 248 [GenBank:HSM_1732]

hypothetical protein [GenBank:HSM_1733] 1978360 1978878 172 [GenBank:HSM_1733]

TetR family transcriptional regulator 1978881 1979504 207 [GenBank:HSM_1734]

major facilitator transporter 1979596 1980798 400 [GenBank:HSM_1735]

small multidrug resistance protein 1980899 1981231 110 [GenBank:HSM_1736]

MarR family transcriptional regulator 1981270 1981719 149 [GenBank:HSM_1737]

hypothetical protein [GenBank:HSM_1738] 1981742 1982113 123 [GenBank:HSM_1738]

hypothetical protein [GenBank:HSM_1739] 1982332 1983432 366 [GenBank:HSM_1739]

cation efflux protein 1983604 1984218 204 [GenBank:HSM_1740]

MerR family transcriptional regulator 1984290 1984688 132 [GenBank:HSM_1741]

hypothetical protein [GenBank:HSM_1742] 1984727 1984873 48 [GenBank:HSM_1742]

hypothetical protein [GenBank:HSM_1743] 1984910 1985062 50 [GenBank:HSM_1743]

alcohol dehydrogenase 1985115 1985723 202 [GenBank:HSM_1744]

putative phage repressor 2124957 2125664 235 [GenBank:HSM_1869]

putative transcriptional regulator, Nlp 2125849 2126118 89 [GenBank:HSM_1870]

transposase 2126129 2128087 652 [GenBank:HSM_1871]

putative transposase 2129292 2130413 373 [GenBank:HSM_1874]

hypothetical protein [GenBank:HSM_1875] 2130790 2131287 165 [GenBank:HSM_1875]

hypothetical protein [GenBank:HSM_1877] 2132108 2133055 315 [GenBank:HSM_1877]

hypothetical protein [GenBank:HSM_1878] 2133039 2133500 153 [GenBank:HSM_1878]

hypothetical protein [GenBank:HSM_1879] 2133840 2134235 131 [GenBank:HSM_1879]

N-6 DNA methylase 2134346 2135887 513 [GenBank:HSM_1880]

hypothetical protein [GenBank:HSM_1881] 2135847 2136245 132 [GenBank:HSM_1881]

hypothetical protein [GenBank:HSM_1882] 2136356 2136550 64 [GenBank:HSM_1882]

hypothetical protein [GenBank:HSM_1886] 2138784 2139098 104 [GenBank:HSM_1886]

transposase 2139228 2139476 82 [GenBank:HSM_1887]

ATPase central domain-containing protein 2139936 2141045 369 [GenBank:HSM_1888]

peptidase S8 and S53 subtilisin kexin sedolisin 2141042 2143234 730 [GenBank:HSM_1889]

hypothetical protein [GenBank:HSM_1890] 2143369 2143800 143 [GenBank:HSM_1890]

cyclase family protein 2160533 2161207 224 [GenBank:HSM_1907]

hypothetical protein [GenBank:HSM_1908] 2161209 2163008 599 [GenBank:HSM_1908]
