# Supplementary material for: Horizontal gene transfer in Histophilus somni and its role in the evolution of pathogenic strain 2336, as determined by comparative genomic analyses
Source: BMC Genomics. 2011 Nov 23;12:570. doi: 10.1186/1471-2164-12-570 (PMC3339403; doi:10.1186/1471-2164-12-570)
Supplement: Additional file 2 — List of H. somni strain 129Pt specific genes. This table lists the strain-specific genes found in H. somni strain 129Pt. This data was obtained by cross-comparison of the genomes of strains 129Pt and 2336 using blastn. [file 1471-2164-12-570-S2.DOCX]

**Additional file 2. List of *H. somni* strain 129Pt specific genes**

**Product Name**  **Start**  **End**  **Length**  **Locus_tag**

hypothetical protein [GenBank:HS_0017a] 18540 18716 58 [GenBank:HS_0017a]

hypothetical protein [GenBank:HS_0099] 92709 92966 85 [GenBank:HS_0099]

transcriptional regulator 93052 93987 311 [GenBank:HS_0100]

hypothetical protein [GenBank:HS_0103] 95537 95761 74 [GenBank:HS_0103]

hypothetical protein [GenBank:HS_0180] 159556 159843 95 [GenBank:HS_0180]

hypothetical protein [GenBank:HS_0213] 215433 215996 187 [GenBank:HS_0213]

hypothetical protein [GenBank:HS_0214] 216015 216398 127 [GenBank:HS_0214]

hypothetical protein [GenBank:HS_0215] 216401 216868 155 [GenBank:HS_0215]

hypothetical protein [GenBank:HS_0216] 217020 218141 373 [GenBank:HS_0216]

hypothetical protein [GenBank:HS_0217] 218189 218512 107 [GenBank:HS_0217]

hypothetical protein [GenBank:HS_0342a] 346369 346554 61 [GenBank:HS_0342a]

hypothetical protein [GenBank:HS_0373] 382622 382969 115 [GenBank:HS_0373]

hypothetical protein [GenBank:HS_0374] 383108 383317 69 [GenBank:HS_0374]

transcriptional regulator 451591 452031 146 [GenBank:HS_0422]

phage-related protein 452273 452509 78 [GenBank:HS_0423]

phage integrase 452512 453636 374 [GenBank:HS_0424]

hypothetical protein [GenBank:HS_0425] 453626 453988 120 [GenBank:HS_0425]

phage DNA-polymerase or DNA-primase 454096 455007 303 [GenBank:HS_0426]

virulence-associated protein E 454967 456397 476 [GenBank:HS_0427]

hypothetical protein [GenBank:HS_0428] 456719 457060 113 [GenBank:HS_0428]

hypothetical protein [GenBank:HS_0429] 457204 457629 141 [GenBank:HS_0429]

restriction enzyme, alpha subunit 457622 459592 656 [GenBank:HS_0430]

restriction enzyme, beta subunit 459573 460649 358 [GenBank:HS_0431]

restriction enzyme, beta subunit 460618 461151 177 [GenBank:HS_0432]

hypothetical protein [GenBank:HS_0433] 461158 462090 310 [GenBank:HS_0433]

PTS system, cellobiose-specific IIC component 464465 465805 446 [GenBank:HS_0437]

ribosomal small subunit pseudouridine 465880 466239 119 [GenBank:HS_0438]

synthase A

hypothetical protein [GenBank:HS_0439] 466559 466792 77 [GenBank:HS_0439]

resolvase/integrase-like protein 468080 468301 73 [GenBank:HS_0442]

ParA-like protein 468440 469108 222 [GenBank:HS_0443]

hypothetical protein [GenBank:HS_0444] 469110 469553 147 [GenBank:HS_0444 ]

resolvase/integrase-like protein 469567 470145 192 [GenBank:HS_0445]

hypothetical protein [GenBank:HS_0446] 470536 471993 485 [GenBank:HS_0446]

hypothetical protein [GenBank:HS_0447] 472432 472653 73 [GenBank:HS_0447]

hypothetical protein [GenBank:HS_0447a] 472874 473056 60 [GenBank:HS_0447a]

hypothetical protein [GenBank:HS_0451] 481084 482853 589 [GenBank:HS_0451]

hypothetical protein [GenBank:HS_0452] 482850 483539 229 [GenBank:HS_0452]

hypothetical protein [GenBank:HS_0453] 483539 484348 269 [GenBank:HS_0453]

hypothetical protein [GenBank:HS_0520] 564106 564357 83 [GenBank:HS_0520]

hypothetical protein [GenBank:HS_0521] 564367 564879 170 [GenBank:HS_0521]

hypothetical protein [GenBank:HS_0526] 566252 566476 74 [GenBank:HS_0526]

hypothetical protein [GenBank:HS_0529] 567352 567585 77 [GenBank:HS_0529]

hypothetical protein [GenBank:HS_0530] 567578 567829 83 [GenBank:HS_0530]

hypothetical protein [GenBank:HS_0531] 567813 568184 123 [GenBank:HS_0531]

hypothetical protein [GenBank:HS_0532] 568181 568708 175 [GenBank:HS_0532]

phage DNA primase-like protein 568711 570621 636 [GenBank:HS_0533]

hypothetical protein [GenBank:HS_0536] 572003 572296 97 [GenBank:HS_0536]

hypothetical protein [GenBank:HS_0538] 572889 573335 148 [GenBank:HS_0538]

hypothetical protein [GenBank:HS_0539] 573579 573782 67 [GenBank:HS_0539]

phage P1-related protein 592009 592530 173 [GenBank:HS_0557]

hypothetical protein [GenBank:HS_0558] 592523 593221 232 [GenBank:HS_0558]

hypothetical protein [GenBank:HS_0567a] 604730 604966 78 [GenBank:HS_0567a]

hypothetical protein [GenBank:HS_0621] 667778 668578 266 [GenBank:HS_0621]

hypothetical protein [GenBank:HS_0622] 668613 669866 417 [GenBank:HS_0622]

membrane protein 672127 673131 334 [GenBank:HS_0625]

hypothetical protein [GenBank:HS_0626] 673133 673495 120 [GenBank:HS_0626]

hypothetical protein [GenBank:HS_0627] 673507 674157 216 [GenBank:HS_0627]

hypothetical protein [GenBank:HS_0628] 674167 674805 212 [GenBank:HS_0628]

hypothetical protein [GenBank:HS_0629] 674853 675530 225 [GenBank:HS_0629]

hypothetical protein [GenBank:HS_0630] 675539 676570 343 [GenBank:HS_0630]

hypothetical protein [GenBank:HS_0631] 676573 678123 516 [GenBank:HS_0631]

serine/threonine kinase-like protein 678116 679612 498 [GenBank:HS_0632]

stress response protein 679642 680220 192 [GenBank:HS_0633]

stress response protein 680223 680969 248 [GenBank:HS_0634]

stress response protein 680979 681560 193 [GenBank:HS_0635]

glycosyltransferase family 8 lipopolysaccharide 682438 683502 354 [GenBank:HS_0636]

biosynthesis protein

hypothetical protein [GenBank:HS_0726] 780444 780668 74 [GenBank:HS_0726]

hypothetical protein [GenBank:HS_0744] 799472 799891 139 [GenBank:HS_0744]

hypothetical protein [GenBank:HS_0745] 799904 800155 83 [GenBank:HS_0745]

hypothetical protein [GenBank:HS_0798] 869335 869568 77 [GenBank:HS_0798]

hypothetical protein [GenBank:HS_0869] 949454 949846 130 [GenBank:HS_0869]

hypothetical protein [GenBank:HS_0917a] 1012275 1012490 71 [GenBank:HS_0917a]

hypothetical protein [GenBank:HS_0936] 1035431 1036189 252 [GenBank:HS_0936]

hypothetical protein [GenBank:HS_0937] 1036481 1037227 248 [GenBank:HS_0937]

hypothetical protein [GenBank:HS_0965] 1067418 1067642 74 [GenBank:HS_0965]

translation initiation factor 1 (eIF-1/SUI1) 1071929 1072246 105 [GenBank:HS_0972]

hypothetical protein [GenBank:HS_1005] 1106463 1106660 65 [GenBank:HS_1005]

lysine/cadaverine antiporter 1107112 1108428 438 [GenBank:HS_1006]

L-lysine decarboxylase 1108445 1110571 708 [GenBank:HS_1007]

chromosome replication initiation 1112316 1113203 295 [GenBank:HS_1009]

inhibitor protein

hypothetical protein [GenBank:HS_1040] 1147178 1147396 72 [GenBank:HS_1040]

transposase 1252323 1252976 217 [GenBank:HS_1116]

hypothetical protein [GenBank:HS_1119] 1255499 1255684 61 [GenBank:HS_1119]

hypothetical protein [GenBank:HS_1199] 1354473 1354667 64 [GenBank:HS_1199]

hypothetical protein [GenBank:HS_1224] 1385418 1385618 66 [GenBank:HS_1224]

integrase 1388586 1389104 172 [GenBank:HS_1228]

hypothetical protein [GenBank:HS_1246] 1427713 1428369 218 [GenBank:HS_1246]

autotransporter protein YapE 1428263 1430725 820 [GenBank:HS_1247]

hypothetical protein [GenBank:HS_1260] 1447281 1448399 372 [GenBank:HS_1260]

hypothetical protein [GenBank:HS_1331] 1525271 1525699 142 [GenBank:HS_1331]

hypothetical protein [GenBank:HS_1332] 1525748 1525927 59 [GenBank:HS_1332]

hypothetical protein [GenBank:HS_1333] 1525943 1526275 110 [GenBank:HS_1333]

terminase small subunit 1526268 1526666 132 [GenBank:HS_1334]

prophage CP4-57 regulatory protein 1526741 1526947 68 [GenBank:HS_1335]

hypothetical protein [GenBank:HS_1336] 1527311 1528012 233 [GenBank:HS_1336]

integrase 1528094 1529350 418 [GenBank:HS_1337]

hypothetical protein [GenBank:HS_1371] 1562582 1563130 182 [GenBank:HS_1371]

hypothetical protein [GenBank: HS_1383] 1580465 1580851 128 [GenBank:HS_1383]

hypothetical protein [GenBank:HS_1392] 1588836 1589234 132 [GenBank:HS_1392]

hypothetical protein [GenBank:HS_1393] 1589385 1589789 134 [GenBank:HS_1393]

hypothetical protein [GenBank:HS_1397] 1591256 1592215 319 [GenBank:HS_1397]

hypothetical protein [GenBank:HS_1404] 1596026 1596478 150 [GenBank:HS_1404]

hypothetical protein [GenBank:HS_1405] 1596523 1596729 68 [GenBank:HS_1405]

repressor protein 1596868 1597503 211 [GenBank:HS_1406]

hypothetical protein [GenBank:HS_1407] 1597622 1598665 347 [GenBank:HS_1407]

hypothetical protein [GenBank:HS_1408] 1598658 1598921 87 [GenBank:HS_1408]

hypothetical protein [GenBank:HS_1409] 1598911 1599276 121 [GenBank:HS_1409]

hypothetical protein [GenBank:HS_1410] 1599786 1600052 88 [GenBank:HS_1410]

hypothetical protein [GenBank:HS_1411] 1600415 1600603 62 [GenBank:HS_1411]

hypothetical protein [GenBank:HS_1412] 1600832 1601518 228 [GenBank:HS_1412]

hypothetical protein [GenBank:HS_1413] 1601521 1601718 65 [GenBank:HS_1413]

hypothetical protein [GenBank:HS_1414] 1602088 1602576 162 [GenBank:HS_1414]

prophage antirepressor 1602930 1603595 221 [GenBank:HS_1415]

phage recombinase 1604708 1605643 311 [GenBank:HS_1419]

hypothetical protein [GenBank:HS_1420] 1605636 1606304 222 [GenBank:HS_1420]

hypothetical protein [GenBank:HS_1421] 1606354 1607412 352 [GenBank:HS_1421]

hypothetical protein [GenBank:HS_1423] 1608571 1609368 265 [GenBank:HS_1423]

phage transcriptional regulator, AlpA 1609378 1609563 61 [GenBank:HS_1424]

integrase 1609525 1610766 413 [GenBank:HS_1425]

hypothetical protein [GenBank:HS_1471] 1664281 1664481 66 [GenBank:HS_1471]

hypothetical protein [GenBank:HS_1512] 1706889 1707977 362 [GenBank:HS_1512]

hypothetical protein [GenBank:HS_1527] 1726631 1727497 288 [GenBank:HS_1527]

hypothetical protein [GenBank:HS_1528] 1727606 1727926 106 [GenBank:HS_1528]

hypothetical protein [GenBank:HS_1529] 1728121 1728684 187 [GenBank:HS_1529]

hypothetical protein [GenBank:HS_1530] 1728711 1728974 87 [GenBank:HS_1530]

hypothetical protein [GenBank:HS_1563] 1761577 1762494 305 [GenBank:HS_1563]

hypothetical protein [GenBank:HS_1568] 1773789 1774133 114 [GenBank:HS_1568]

hypothetical protein [GenBank:HS_1660] 1890486 1890689 67 [GenBank:HS_1660]

hypothetical protein [GenBank:HS_1676] 1906732 1907013 93 [GenBank:HS_1676]

hypothetical protein [GenBank:HS_1677] 1907010 1907219 69 [GenBank:HS_1677]

hypothetical protein [GenBank:HS_1680] 1909106 1909975 289 [GenBank:HS_1680]

hypothetical protein [GenBank:HS_1680] 1909106 1909975 289 [GenBank:HS_1680]

NAD-dependent aldehyde dehydrogenase 1910070 1910312 80 [GenBank:HS_1681]

aldehyde dehydrogenase 1910279 1910476 65 [GenBank:HS_1682]

hypothetical protein [GenBank:HS_1726] 1960349 1961146 265 [GenBank:HS_1726]

hypothetical protein [GenBank:HS_1727] 1961150 1961359 69 [GenBank:HS_1727]

hypothetical protein [GenBank:HS_1728] 1961363 1961557 64 [GenBank:HS_1728]

hypothetical protein [GenBank:HS_1730] 1962274 1962732 152 [GenBank:HS_1730]

hypothetical protein [GenBank:HS_1731] 1962695 1963465 256 [GenBank:HS_1731]

hypothetical protein [GenBank:HS_1733] 1964537 1965055 172 [GenBank:HS_1733]

hypothetical protein [GenBank:HS_1734] 1965052 1965303 83 [GenBank:HS_1734]

hypothetical protein [GenBank:HS_1736] 1966253 1966693 146 [GenBank:HS_1736]

hypothetical protein [GenBank:HS_1740] 1970520 1970813 97 [GenBank:HS_1740]

hypothetical protein [GenBank:HS_1741] 1971203 1971508 101 [GenBank:HS_1741]

hypothetical protein [GenBank:HS_1742] 1971652 1972299 215 [GenBank:HS_1742]

hypothetical protein [GenBank:HS_1743] 1972320 1972517 65 [GenBank:HS_1743]

hypothetical protein [GenBank:HS_1745] 1973220 1973666 148 [GenBank:HS_1745]

hypothetical protein [GenBank:HS_1749] 1975888 1976151 87 [GenBank:HS_1749]

hypothetical protein [GenBank:HS_1754] 1980104 1980292 62 [GenBank:HS_1754]

hypothetical protein [GenBank:HS_1755] 1980289 1980570 93 [GenBank:HS_1755]

hypothetical protein [GenBank:HS_1768] 1990896 1991228 110 [GenBank:HS_1768]

hypothetical protein [GenBank:HS_1769] 1991256 1991651 131 [GenBank:HS_1769]

hypothetical protein [GenBank:HS_1770] 1991657 1991989 110 [GenBank:HS_1770]

hypothetical protein [GenBank:HS_1771] 1992060 1992461 133 [GenBank:HS_1771]

hypothetical protein [GenBank:HS_1772] 1992461 1992700 79 [GenBank:HS_1772]

hypothetical protein [GenBank:HS_1777] 1996990 1997430 146 [GenBank:HS_1777]

hypothetical protein [GenBank:HS_1779] 1997780 1998025 81 [GenBank:HS_1779]

hypothetical protein [GenBank:HS_1782] 2000019 2000252 77 [GenBank:HS_1782]

hypothetical protein [GenBank:HS_1783] 2000602 2001069 155 [GenBank:HS_1783]

hypothetical protein [GenBank:HS_1784] 2001071 2001334 87 [GenBank:HS_1784]

hypothetical protein [GenBank:HS_1787] 2002226 2003155 309 [GenBank:HS_1787]

hypothetical protein [GenBank:HS_1788] 2003166 2003639 157 [GenBank:HS_1788]

hypothetical protein [GenBank:HS_1790] 2004189 2004617 142 [GenBank:HS_1790]

hypothetical protein [GenBank:HS_1791] 2004619 2005728 369 [GenBank:HS_1791]

hypothetical protein [GenBank:HS_1792] 2006035 257 640 [GenBank:HS_1792]
